# SYNAPTOTAGMIN 4 is expressed mainly in the phloem and participates in abiotic stress tolerance in Arabidopsis

**DOI:** 10.3389/fpls.2024.1363555

**Published:** 2024-07-01

**Authors:** Ajay Kumar, Miroslav Krausko, Ján Jásik

**Affiliations:** Department of Experimental Plant Biology, Institute of Botany, Plant Science and Biodiversity Centre, Slovak Academy of Sciences, Bratislava, Slovakia

**Keywords:** Arabidopsis SYT4, phloem, gene expression, root cap, insertion mutants, stress response

## Abstract

Plant synaptotagmins structurally resemble animal synaptotagmins and extended-synaptotagmins. Animal synaptotagmins are well-characterized calcium sensors in membrane trafficking, and extended-synaptotagmins mediate lipid transfer at the endoplasmic reticulum—plasma membrane contact sites. Here, we characterize *SYNAPTOTAGMIN 4 (SYT4)*, which belongs to the six-member family in Arabidopsis. Fluorometric GUS assay showed that the *SYT4* promoter was strongest in roots and the least active in rosettes and cauline leaves, which was confirmed by qPCR. In seedlings, promoter activity was influenced by several factors, such as plant growth regulators, mannitol, sucrose, polyethylene glycol and cold. GUS histochemistry revealed *SYT4* promoter activity in the phloem of all organs and even almost exclusively in sieve element precursors and differentiating sieve elements. Accordingly, the SYT-GFP fusion protein also accumulated in these cells with maximal abundance in sieve element precursors. The protein formed a network in the cytoplasm, but during sieve tube differentiation, it deposited at the cell periphery and disappeared from mature tubes. Using photoconvertible fluorescence technology, we showed that a high abundance of SYT4 protein in meristematic protophloem cells was due to its extensive synthesis. SYT4 protein synthesis was interrupted in differentiating sieve elements, but protein degradation was also reduced. In addition to phloem, the fusion protein was detected in shoot and root stem cell niche as early as the late heart stage of the embryo. We isolated and molecularly and biologically characterized five *syt4* T-DNA insertion alleles and subjected them to phenotype analysis. The allele with the C2B domain interrupted by an T-DNA insertion exhibits increased sensitivity to factors such as auxins, osmotics, salicylic acid, sodium chloride, and the absence of sucrose in the root growth test.

## 1 Introduction

Synaptotagmin I in animals was discovered as p65 using a monoclonal antibody labeling synaptic vesicles (Matthew et al., 1981). Later, many Synaptotagmin (SYT) isoforms were identified and shown to be involved in diverse cellular functions in different tissues (Wolfes and Dean, 2020). SYTs contain two calcium-binding C2 domains at their C-termini and a single transmembrane domain at the N-termini (Pang and Südhof, 2010). More recently, similar proteins to SYTs, Extended-synaptotagmins (E-SYTs) in mammals and tricalbins in yeast were identified as part of the membrane contact sites (Saheki et al., 2016; Nath et al., 2020; Thomas et al., 2022). They possess an additional region between the C2 tandem and the transmembrane domain named Synaptotagmin-like mitochondrial-lipid-binding domain (Wang et al., 2023).

Plants also contain proteins closely related to animal SYTs and E-SYTs. Arabidopsis, a commonly studied plant model, has five SYT members with the transmembrane domain and two C2 domains (Craxton, 2004, 2010). According to the protein sequence alignment, Arabidopsis SYTs belong to two clades; one includes SYT1, SYT2, and SYT3, and a second, SYT4 and SYT5 (García-Hernández et al., 2024). SYT1 is the most intensively investigated member of the family and has a pivotal role in mitigating different stresses (Benitez-Fuente and Botella, 2023). It was initially identified through mass spectrometry among the plasma membrane proteins of plants acclimatized to cold (Kawamura and Uemura, 2003). Consequently, SYT1 was demonstrated to increase the freezing and salt tolerance in a calcium-dependent manner (Schapire et al., 2008; Yamazaki et al., 2008). Biochemical and physiological analyses indicated that SYT1 might function as an electrostatic phospholipid anchor, conferring mechanical stability in plant cells (Pérez-Sancho et al., 2015). Further, SYT1 was demonstrated to be involved in ionic stress responses (Lee et al., 2019) and heat tolerance by reducing lipid peroxidation (Yan et al., 2017). Several lines of evidence exist for the involvement of SYT1 in responses to biotic stresses. It reduced the amount of PENETRATION1 (PEN1) syntaxin at the post-translational level, and this reduction affects the formation of the ternary SNARE complex, which ultimately influences plant resistance against powdery mildew fungi (Kim et al., 2016). *SYT1* is well-documented to facilitate virus genome transportation through plasmodesmata (Lewis and Lazarowitz, 2010; Uchiyama et al., 2014). The protein recognizes the plasmodesmata localization signal on virus proteins and facilitates their attachment with the plasmodesmata membrane (Yuan et al., 2018). Recently, we have shown that mutation in *SYT1* increases the adverse effect of salt stress on photosynthesis, a critical physiological process in plants (Krausko et al., 2022).

The functions of other family members are poorly understood. SYT2, the closest homolog of SYT1, is localized at the PM and in Golgi (Zhang et al., 2011; Wang et al., 2015). It is specific for gametophytes and required for pollen germination and pollen tube growth (Wang et al., 2015). SYT3 and SYT5 probably have a redundant function with SYT1 (Lee et al., 2020; Ruiz-Lopez et al., 2021). SYT5 has been shown to promote the interaction between SYNTAXIN OF PLANTS 123 (SYP123) and VESICLE ASSOCIATED MEMBRANE PROTEIN721/722 (VAMP721/722), thereby reducing the proliferation of *Pseudomonas syringae* bacteria (Kim et al., 2021).

The mechanisms by which SYTs are involved in plant stress responses have been extensively addressed. It was demonstrated to help in resealing the damaged membrane (Schapire et al., 2008; Yamazaki et al., 2008). Recent studies revealed that plant SYTs may act as animal E-SYTS (Benitez-Fuente and Botella, 2023 for review). Studies were performed mainly on epidermal cotyledon cells. SYT1 and also SYT3 and SYT5 are enriched at the endoplasmic reticulum (ER) and plasma membrane (PM) contact sites along with other proteins such as NETWORKED 3C (NETC3), SYP123, VAMP721/722, VESICLE ASSOCIATED PROTEIN27 (VAP27; Pérez-Sancho et al., 2015; Siao et al., 2016; Ishikawa et al., 2018; Lee et al., 2019, Lee et al., 2020; Ruiz-Lopez et al., 2021). According to a recent concept, SYTs help transport diacylglycerol from PM to ER to prevent PM damage (Lee et al., 2019, Lee et al., 2020; Benavente et al., 2021).

SYT4 is the least characterized family member in Arabidopsis. Only Kim et al. (2022) proposed that it has an additive function to SYT5 in Pseudomonas infection. However, a detailed characterization of the gene has not been accomplished. This study aimed to delineate the spatio-temporal expression patterns of *SYT4* and to uncover possible developmental and physiological consequences of the absence of function of this gene under normal and stressful conditions. We have found that *SYT4* is expressed in the phloem of all organs, specifically in sieve element (SE) precursors and developing sieve tubes. In addition, the protein was detected in shoot and root stem cell niche. We characterized five insertional mutants and showed that, under *in vitro* conditions, the *syt4–3* allele exhibits increased sensitivity to factors such as auxins, osmotics, salicylic acid, sodium chloride, or the absence of sucrose.

## 2 Materials and methods

### 2.1 Plant material and growth conditions

*Arabidopsis thaliana* (Col-0) wild-type, transgenic and mutant plants were cultivated in pots with soil substrate (50% peat moss, 30% perlite and 20% sand) under a temperature of 22°C, humidity of 40% - 60% and 14 h light provided by white-colored LED panels at an intensity of 150 µmol m^-2^ s^−1^/10 h dark photoperiod. Seeds were surface sterilized with 70% ethanol for 1 min and then with 1% sodium hypochlorite for 20 min. After washing three times with distilled water, seeds were sown in Petri dishes on standard cultivation medium (SCM, 1/2 MS basal salts, 0.4 mg L^–1^ thiamine HCl, 100 mg L^–1^ myo-inositol, 10 g L^–1^ sucrose, 7 g L^–1^ agar). Plates were cold stratified at 4°C for 3 days, then moved to the cultivation chamber and kept under conditions mentioned above.

### 2.2 DNA technology, plant transformation and transgenic lines creation

Genomic DNA was isolated from young rosette leaves using the innuPREP plant DNA kit (Analytik Jena, Jena, Germany) or with the CTAB method (Rogers and Bendich, 1989). For preparing the *pSYT4-GUS* transcriptional fusion construct, a 2,053-bp-long DNA stretch located upstream of the start codon was amplified by PCR from genomic DNA and inserted into the *pPCV812* vector (Koncz et al., 1994) between the XhoI and BamHI restriction sites. For protein localization studies, we prepared *pSYT4::SYT4::Dendra2::pASYT4* (hereafter abbreviated as *SYT4-Dendra2*) and *pSYT4::SYT4::EGFP::pASYT4* (*SYT4-GFP*) transcriptional-translational fusion constructs. Genomic *SYT4*, with a 2,087 bp long DNA sequence located upstream of the start codon and a 485 bp long DNA stretch located downstream of the stop codon, was amplified by PCR from genomic DNA of Arabidopsis (Col-0). Dendra2 was amplified from *p35S::Dendra2* (Jásik et al., 2013) and EGFP from *pCS2 + EGFP-NB* plasmids (Boggetti et al., 2012). Fragments were sequentially inserted into the *pAMPAT-MSC* vector (GenBank: *AY436765.1*) between the AscI and NotI sites. DNA stretches for *Dendra2* and *EGFP* were attached to the 3′ end of the *SYT4* genomic DNA sequence from which the stop codon was removed. All fragments were amplified using Q5^®^ Hot Start High-Fidelity DNA Polymerase and primers available in **Supplementary Table 1**. All enzymes were purchased from New England BioLabs (Frankfurt, Germany). The correctness of sequences and read frames were verified by sequencing. Plasmids were transferred into the GV3101 (pMP90RK) *Agrobacterium tumefaciens* strain (Koncz and Schell, 1986), and this was used to transform *Arabidopsis thaliana* (Col-0) using the floral-dip method (Clough and Bent, 1998). The transformation was performed twice, and eight pots containing 5–8 Arabidopsis plants were used for each construct. Selection of primary transformants was carried out on the standard cultivation medium with 7.5 mg L^-1^ phosphinothricin for the *SYT4-GFP* and *SYT4-Dendra2* constructs and with 15 mg L^-1^ hygromycin for the *pSYT4-GUS* construct. Both chemicals were purchased from Duchefa (Haarlem, The Netherlands). Based on the resistance, we selected 31 plants for *pSYT4-GUS*, 87 plants for *SYT4-GFP*, and 54 plants for the *SYT4-Dendra2* construct. The expression of the fusion proteins was verified in offspring seedlings under an epifluorescence microscope, and the activity of the *SYT4* promoter was monitored using a GUS histochemical assay. We then subjected the lines with signals to segregation analysis based on resistance (see above) to eliminate lines with multiple T-DNA inserts. We finally selected homozygous lines with a single insertion and obtained 11 homozygous lines with one insertion for *pSYT4-GUS*, 10 for *SYT4-GFP*, and 7 for *SYT4-Dendra2*. The presence of correct fusions in transgenic lines was verified by PCR. The *SYT1-Dendra2* line was described previously (Lešková et al., 2019, Lešková et al., 2020).

### 2.3 GUS histochemical and fluorometric assays

Promoter activity in different organs was estimated using *pSYT4::GUS* transgenic plants in combination with histochemistry and fluorometry described previously (Jásik et al., 2011). For whole-mount histochemistry, seedlings growing *in vitro* were incubated in X-GlcA reagent mixture (1 mM 5-Bromo-4-chloro-3-indolyl-β-D-glucuronic acid [Duchefa, Haarlem, Netherlands] dissolved in 50 mM phosphate buffer (pH 7.3) supplemented with 10 mM EDTA, 2 mM ferrocyanide, 2 mM ferricyanide, 0.1% Triton X-100 [v/v] and 10% methanol). Samples were kept in the dark at 37°C. Blue-stained samples were treated with 70% ethanol to remove the chlorophyll and mounted to 80% glycerol for overall examination. Alternatively, after the GUS procedure, seedling parts were fixed with 4% glutaraldehyde, embedded in Stedman wax (Vitha et al., 2000), and sectioned. After dewaxing in ethanol, sections were stained with 0.1% basic fuchsine. GUS activity in complex organs of plants grown in the pots was analyzed by histochemistry, also using cryosections as described by Krausko et al. (2021). Shortly, cryosections collected in microcentrifuge tubes in cryostat were washed with MTSB, then treated with X-GlcA reagent mixture, and stained with 0.1% basic fuchsine. Sections were then washed and embedded in glycerol on microscopic slides. For the GUS fluorometry assay, samples of different organs of plants cultivated in the pots in growth stage 6.50 (50% of flowers to be produced have opened, Boyes et al., 2001) were homogenized in protein extraction buffer (50 mM phosphate buffer, 10 mM EDTA, 0.1% Triton X-100, 0.1% SDS) in 1.5 mL Eppendorf microcentrifuge tubes with LLG metal micro pestle fitted in a hand drill. Extracts were centrifuged at 16,000 x g and 4°C, and protein concentration in the supernatant was determined with a DC protein assay kit (Bio-Rad, Hercules, California, USA). Afterward, samples were diluted with extraction buffer to equal protein concentration. The reaction was carried out in 50 µL extraction buffer containing 10 µg total proteins and 2 mM 4-Methylumbelliferyl-ß-D-glucuronide trihydrate (4-MUG, Duchefa, Haarlem, Netherlands) in a black 96 well-plate (BRAND, Wertheim, Germany). The plates were incubated in the dark at 37°C, and after 2 h, the reaction was stopped with 200 μL 0.2 M Na_2_CO_3_. Fluorescence signal intensity was determined using a microplate reader Fluoroskan Ascent^®^ FL (Thermo Fisher Scientific, Waltham, USA), employing 355 nm excitation and 485 nm emission filters. The promoter activity in seedlings influenced by various factors was determined using the GUS fluorometric technique. Seedlings were grown on SCM in the growth chamber at 22°C under continuous light with 100 µmol m^-2^ s^-1^ light intensity. Petri dishes were kept in a vertical position. Ten-day-old seedlings were transferred to new SCM supplemented with different substances or kept under different conditions. After 24 h and 48 h, the roots and aboveground parts were separated, and intact samples were placed in 96-well microplates containing a 150 µl fluorometry extraction buffer with 2 mM MUG substrate. The microplates with samples were incubated in the dark for two hours at 37°C. The reaction was then stopped by adding 150 µl of 0.2 M Na_2_CO_3_, and fluorescence signal intensity was recorded as mentioned above.

### 2.4 RNA isolation, cDNA synthesis and RT-PCR

For analysis of overall transcript abundance, total RNA was obtained from different parts of plants in growth stage 6.50, as characterized previously (Boyes et al., 2001). For transcript estimation in mutant alleles, RNA was isolated from roots or aboveground parts of seedlings originating from homozygous mutant plants (obtained as described below). RNA was isolated using the RNeasy plant mini kit (Qiagen, Hilden, Germany). DNA contamination was removed with a TURBO DNA-free™ kit (Thermo Fisher Scientific, Waltham, MA, USA). First-strand cDNA for transcript abundance analysis in different organs was synthesized from 500 ng of total RNA using a FIREScript® RT cDNA synthesis KIT (Solis BioDyne, Estonia) and a mix of oligo-dT and random primers. The first-strand cDNA for transcript analysis in mutants was synthesized using the same kit employing oligo-dT or oligo-dT and a random primer mix. Alternatively, cDNA was prepared using SOLIScript® RT cDNA synthesis KIT (Solis BioDyne, Estonia) and the gene-specific primer for *SYT4* (**Supplementary Table 1**).

Transcript abundance estimation in six different organs and mutant alleles (characterized below) was performed on Bio-Rad CFX Connect real-time PCR detection system with SsoAdvanced™ universal SYBR® green supermix. We carried out four independent biological replicates and two technical replicates. *PROTEIN PHOSPHATASE 2A SUBUNIT A3* (*PP2A*, *AT1G13320*), *MONENSIN SENSITIVITY1* (*MON1*, *AT2G2839*0) and *POLYUBIQUITIN 10* (*UBQ10*, *AT4G05320*) shown previously to have expression stability throughout development and under a range of environmental conditions (Czechowski et al., 2005) were used for normalization of gene expression. Sequences of primers are available in **Supplementary Table 1**. Results were analyzed using the Bio-Rad CFX Manager 3.1 software, and expressions were quantified through the ΔCt method. Differences in relative expression between the organs were estimated through one-way ANOVA and Student's t-test. Differences in the abundance of transcript in roots of mutant alleles were evaluated also by qPCR as mentioned above. Additionally, we tested the presence of transcripts in roots and aboveground parts of seedlings of the mutant alleles using different combinations of primers and c-DNAs by semi-quantitative RT-PCR. Sequences of primers used in testing are listed in **Supplementary Table 1**.

### 2.5 Identification of *syt4* mutants and analysis of T-DNA insert junctions

We purchased five alleles from The European Arabidopsis Stock Centre (NASC: http://arabidopsis.info/). SALK_201787C (named here *syt4–1*) was from the Salk collection (Alonso et al., 2003), two alleles SAILseq_652_E05.1 (*syt4–2*) and SAIL_359_H05 (*syt4–3*) from the Syngenta population (Sessions et al., 2002), and two alleles GK-215E11 (*syt4–4*) and GK668A12 *(syt4–5)* from Gabi-Kat collection (Rosso et al., 2003). Syngenta seeds for both alleles were sown in pots, and next-generation seedlings were subjected to segregation analysis using 7,5 mg/L phosphinothricin. Two lines for each allele showing resistance of all seeds were genotyped by PCR. We used combinations of gene-specific primers located upstream and downstream of expected insertion sites and SAIL-LB2 T-DNA insert-specific primers. Fragments were sequenced with the SAIL-LBJJ primer. Original seeds of the Salk *syt4–1* allele have been sown in pots, and individual plants were genotyped with the SALK-LBJJ insert-specific primer and gene-specific primers. PCR fragments were sequenced with the SALK-LBb1.3 primer. Seeds of the *syt4–4* and *syt4–5* alleles were subjected to segregation analysis on SCM with 7.5 mg L^-l^ sulfadiazine. Two lines for each allele showing resistance of all seedlings were genotyped using combinations of gene-specific and GABI-LBJJ and GABI-RBJJ insert-specific primers. The PCR products obtained with GABI-LBJJ and gene-specific primers were sequenced with GABI-LBo8409 primer, the product of GABI-RBJJ primer and insert-downstream primer obtained in *syt4–4* lines were sequenced with the GABI-RBo3144/35st primer.

### 2.6 Immunohistology analysis

Different plant organs and seedlings were processed according to Vitha et al. (2000). The samples were fixed with 4% formaldehyde in MTSB for 3 h, washed thrice with the same buffer, and dehydrated with ethanol and then embedded in Steedman’s wax. Six µm thick sections were prepared using a rotary microtome. Section ribs were mounted on glass covered with glycerol albumin and, after drying, dewaxed with ethanol and processed for immunolocalization. Mouse monoclonal antibody against Dendra2 (clone OTI1G6, Origene Tech. Inc., Rockville, MD, dilution 1:200) was combined with in rat grown antibodies against JIM13 (Knox et al., 1991) or LM5 (Jones et al., 1997) (1:100). Donkey anti-Mouse IgG (H+L) antibody, DyLight™ 550 (1:150, ab96876, Abcam, Cambridge, MA, USA) used for Dendra2 and Goat Anti-Rat IgG-FITC (1:150, OB3050–02, SouthernBiotech, Birmingham, AL, USA) for JIM13 and LM5 visualization. Alternatively, we combined goat Anti-Mouse IgG (whole molecule)–FITC antibody (F9006, Sigma-Aldrich) and Goat anti-Rat IgG (H+L), Alexa Fluor® 555 (1:150, A-21434, Invitrogen, Carlsbad, CA, USA).

### 2.7 Confocal microscopy

All examinations were performed with Olympus FV1000 confocal laser scanning microscope. Seedlings were grown as described previously (Jásik et al., 2013; Lešková et al., 2019). The signal of aniline blue, sirofluor and aesculin were excited with a 405 nm laser and collected with 480–495 nm barrier filter, EGFP, the green population of Dendra2, and FITC were excited with an Argon/2 488 nm laser and captured with a 505 to 525 nm barrier filter, red form of Dendra2, basic fuchsine, DyLight™ 550 and Alexa Fluor^®^ 555 were excited with 543 nm laser and signal collected with a 560–620 nm barrier filter. ImageJ software was used to quantify the signal intensity.

### 2.8 Germination evaluation and primary root growth assay

Germination assay was performed with *syt4–2* and *syt4–3* mutants. Seeds of mutants and the SAIL wild type were sown in Petri dishes on SCM with 1% sucrose or without sucrose. After stratification as described above, Petri dishes were moved to permanent light (100 µmol m^-2^ s^-1^, 22°C) and the proportion of seeds with visible radicles was calculated after 24 and 48 h. In addition, the proportion of seedlings with hypocotyl and cotyledons fully emerged from the seed coat was evaluated after 48 h. To study the inhibitory effect of different factors on the elongation of primary roots, seeds of all five insertion mutant alleles and wild types from the same collection as mutant alleles were surface-sterilized and stratified, as described above. Seeds germinated vertically for another three days in permanent light to produce seedlings for experiments. Then, seedlings of the same size with roots approximately 5 mm long were transferred on the media with different factors in square Petri dishes. We tested the effects of IAA (0.1 µM), NAA (0.1 µM), ABA (20 µM), SA (20 µM), BAP (0.5 µM), mannitol (150 mM), NaCl (75 mM) and sucrose (0 and 6%). SCM with 1% sucrose was used as a control. Seedlings of individual mutant alleles were grown in the same Petri dish with corresponding wild-type seedlings. Twenty seedlings were used for each genotype and treatment, and the experiments were repeated minimally three times. Petri dishes were kept in a vertical position under the conditions mentioned above. After 3 days, the seedlings were photographed, and the root length increments were analyzed. Root lengths of mutants and wild-type plants affected by factors were related to root lengths of seedlings growing on SCM.

### 2.9 Data evaluation, processing, and presentation

The figure images were processed by ImageJ, Adobe Photoshop CS2 (Adobe Systems, Mountain View, USA), and Microsoft Publisher software (Microsoft, Redmond, USA). The length of the roots was measured with ImageJ software using a neuroanatomy-SNT plugin. In charts, bars correspond to the standard errors of means. Significances between samples were analyzed by ANOVA and Student's t-test. Values in charts are given as the mean ± SE.

## 3 Results

### 3.1 Quantitative determination of *SYT4* promoter activity in organs

**Figure 1:**
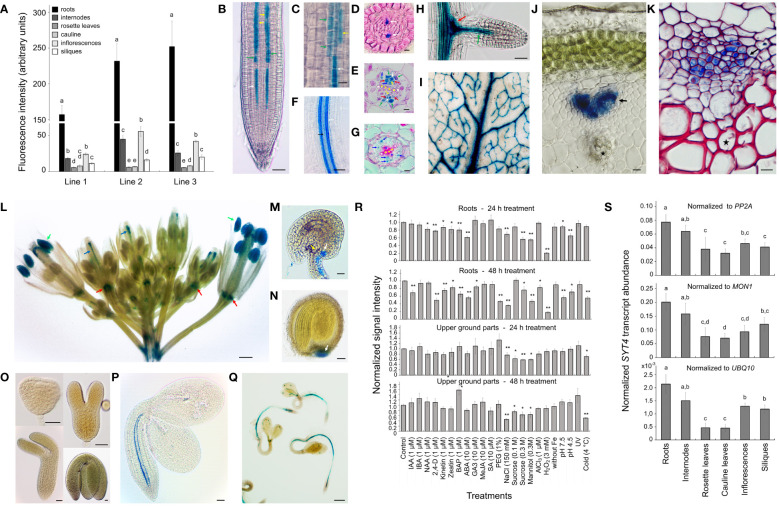
*SYT4* promoter activity and transcript abundance. **(A)** shows the GUS fluorometric analysis of promoter activity in different organs of three homozygous, pot-grown *pSYT4-GUS* transgenic lines with one T-DNA insert in growth stage 6.50 (Boyes et al., 2001; values in columns followed with different letters are significantly different, p ≤ 0.05, n=8). The pattern of *SYT4* promoter activity in the root using whole mount histochemical GUS procedure is in **(B)**. **(C)** shows the root part in the transition zone (300 – 600 µM from QC, asterisk in **B**). Cross sections through the primary root of two-week-old seedlings are at a distance of 80 µM **(D)**, 1 mm **(E)** and 4 mm **(G)** from QC (in **B**–**E** and **G** white arrows point to PP, yellow to MP, red to CC, blue to phloem PC and green to phloem pole pericycle; white asterisks mark protoxylem, yellow metaxylem, blue primary xylem elements). **(F)** shows the root area with a differentiating protoxylem (black arrow) and several files of GUS-positive cells in the phloem (white arrow). In lateral roots, the promoter was very active in clumps of cells at the base of the lateral root primordium (red arrow in **H**) and phloem PC (green arrow). Leaves show GUS activity in the vasculature **(I)**. Young shoots show a strong signal in the phloem pole differentiating from PC (**J**, the reaction was performed on a hand section). In the shoot in the early stage of secondary thickening, different phloem cells show the variable intensity of staining (**K**, whole mount samples treated for GUS detection were fixed and sectioned; the red color is due to basic fuchsine staining). The inflorescence shows intense GUS activity in the anthers (green arrows in **L**), the pistil vasculature (blue arrows), and the flower abscission zone (red arrows). Ovules and seeds are GUS-positive at their chalazal poles **(M, N)**, but no promoter activity was identified during embryogenesis **(O)**. In embryos of seeds imbibed for 24 h, the signal was evident in the root and hypocotyl phloem PC in the form of two files **(P)**. After 72 h, staining was present in the vasculature along the entire seedling body, and several starting points of PP differentiation were observed **(Q)**. **(R)** shows the effects of different factors on promoter activity in roots and aboveground parts estimated by the GUS fluorometry assay (differences between the treated and control samples are indicated by single asterisk for p < 0.05 and double asterisks for p < 0.01, n = 9). **(S)** displays transcript abundance of *SYT4* in different organs analyzed by real-time qPCR (values in columns followed with different letters are significantly different, p ≤ 0.05, n=4).). Size bars – **(C–E, G, J, K, M)** =10 µm; **(O)** = 25 µm; **(B, F, H, I, N, P)** = 50 µm; **(L, Q)** =500 µm.

Since the expression of the *SYT4* gene was not previously characterized, we analyzed it in different organs. We first studied *SYT4* promoter activity using the *uidA* reporter gene that encodes beta-glucuronidase (GUS). Three homozygotic lines with a single copy of the T-DNA insert were used to analyze the activity of the *SYT4* promoter in different organs by GUS fluorometry assay. Plants grew in pots and were in the middle flowering stage (stage 6.50, Boyes et al., 2001). The promoter activity patterns among organs were similar for all three lines; however, the fluorescence values among lines varied considerably. The highest promoter activity was detected in roots, less in inflorescence and stem, followed by siliques, and the lowest promoter activity was identified in both leaf types (**Figure 1A**).

### 3.2 Histochemical analysis of promoter-GUS-reporter lines

Then, we investigated the activity of the promoter in different organs during development by GUS histochemical detection. Using the whole-mount GUS assay, we observed blue staining in vascular bundles of all plant organs (**Figures 1B–L**), and in addition in pollen grains of open flowers (**Figure 1L**), in vasculature at chalazal ends of ovules (**Figure 1M**) and seeds (**Figure 1N**), and floral organ abscission zones (**Figure 1L**). No promoter activity was detectable during embryo development and maturation (**Figure 1O**). However, when we analyzed imbibing seeds, we saw promoter activity in embryo roots and hypocotyls 24 h after sowing (**Figure 1P**). After 72 h, the staining was observable in vascular bundles of whole seedlings (**Figure 1Q**), although coloring was interrupted, especially in the cotyledons. In the seedling root tips, a clear signal was apparent already in isodiametric cells in protophloem (PP) files (**Figures 1B–D**) and more proximally also in incipient metaphloem cells (MP), companion cells (CC), phloem pole pericycle cells and cells of the phloem pole of procambium (PC; **Figures 1B, C, E**). In several transgenic lines (three from eleven with one insertion of T-DNA), we observed intense staining in the root cap (RC; **Supplementary Figure 2A**), but other lines show only a feeble reaction even after extended treatment (24 h). Several GUS-positive cell files were observed in the phloem of the root differentiation zone, and staining intensity varied among files (**Figures 1F, G**). No GUS reaction was detected in the lateral root primordium (**Supplementary Figure 2C**); however, intense staining was seen in emerged lateral roots in their central basal parts and developing PP (**Figure 1H**). In the leaf, the GUS reaction was observed mainly in the primary vein (**Figure 1I**). In the young, just emerging inflorescence stem (developmental stage 5, Boyes et al., 2001), GUS activity was limited to the phloem PC (**Figure 1J**). Secondary thickening stems had positive cells in the phloem area, but cells showed different staining intensities (**Figure 1K**).

### 3.3 Effect of different factors on *SYT4* promoter activity

At first, we analyze the *pSYT4* DNA sequence used in our constructs employing the PlantCARE database of plant cis-acting regulatory elements (Lescot et al., 2002). We identified several cis-regulatory elements (**Supplementary Table 2**). Besides five MYB recognition and binding sites and three MYC cis-elements that are responsive to different stresses, we also found one DRE1 drought responsiveness element, four ABRE abscisic acid-responsive elements, two ARE regulatory element essential for the anaerobic induction, one TGA auxin-responsive element, LTR cis-acting element involved in low-temperature responsiveness and finally the light-responsive regulatory elements such as G-box, AE-box, and TCT-motif.

To understand how the expression of *SYT4* is regulated at the promoter level, we examined the effects of different factors on the promotor activity using GUS quantitative fluorometry. For experiments, we used 10-day-old seedlings that were large enough to produce a sufficient quantity of signal to perform analysis with single intact roots and aboveground parts. Seedlings were exposed to 21 treatments for 24 h and 48 h, and then the roots and aboveground parts were separated and immersed in the MUG reaction solution. Results are summarized in **Figure 1R**. Application of growth regulators, namely NAA, 2,4-D, kinetin, zeatin, BAP, and ABA, for 24 h and 48 h significantly reduced the promoter activity in roots; however, the activity of the promoter in the aboveground parts was unaffected (**Figure 1R**). The exposure of seedlings to osmotics, namely, PEG, sucrose, and mannitol, severely reduced *SYT4* promoter activity in roots and excluded PEG also in aboveground parts (**Figure 1R**). Finally, also NaCl, cold and peroxide treatment dramatically decreased promoter activity in roots and aboveground parts (**Figure 1R**).

Furthermore, we wanted to know whether the overall promoter activity was reduced proportionally in the phloem throughout the root or only in its particular parts. Qualitative histochemical analysis showed that the decrease in promoter activity detected by the fluorometry was mainly due to the absence of promoter activity in the phloem of the matured root region. In contrast, the promoter was still active in the phloem in the root tip. This was evident in the case of 2,4-D (**Supplementary Figure 2F**), mannitol (**Supplementary Figure 2G**), and NaCl (**Supplementary Figure 2H**) treatment. Under NaCl and mannitol stress, we observed remarkable variability of *SYT4* promoter activity among cells in the phloem files. In roots growing on SCM, the *SYT4* promoter activity was not interrupted in matured roots, and the promoter strength did not change so drastically from cell to cell (**Supplementary Figure 2E**, see also **Figure 1B**).

### 3.4 *SYT4* transcript abundance

In the next step, we estimated *SYT4* transcript abundance in different organs of Arabidopsis, as in the case of the promoter, in the middle stage of flowering, as characterized previously (Boyes et al., 2001). We employed qPCR and primers designed to bridge exon 6/7 and exon 7/8 junctions to amplify specifically *SYT4* cDNAs. Values normalized to three commonly used reference genes *PP2A*, *MON1* and *UBQ10* (Czechowski et al., 2005) are shown in **Figure 1S**. The *SYT4* transcript was most abundant in the roots, followed by the stems. Rosette and cauline leaves showed the lowest transcript abundance.

### 3.5 SYT4 protein in embryos and seedlings

**Figure 2:**
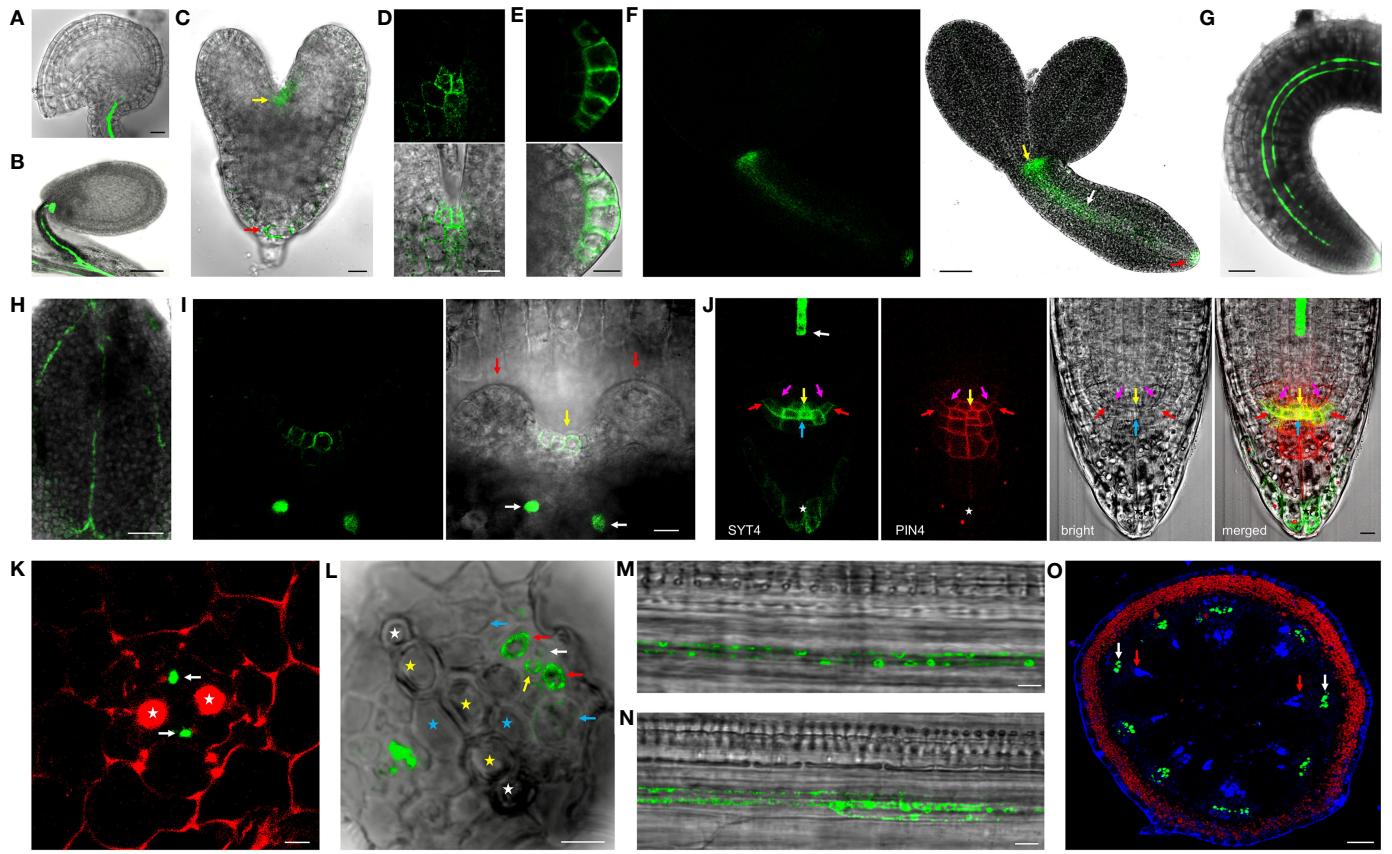
SYT4 protein in embryos and seedlings. In mature ovules **(A)** and developing seeds **(B)**, SYT4-GFP fusion protein is present only in the vasculature of funicules and chalazal poles. In embryos, the fusion protein first appears in both shoot (yellow arrow) and root (red arrow) apical meristem during their late heart-shaped stage (**C**; in **D**, is a close-up view of the shoot, and in **(E)** root apical meristem of this stage embryo), and subsequently in PC during the late torpedo stage (white arrow in **F;** other arrows as in **C**). In germinating embryos (48 h after seed imbibition), the signal is visible in the root and hypocotyl as two files **(G)** and in the midvein and secondary veins in cotyledons **(H)**. The signal is also present in the shoot apical meristem in advanced 6-day-old seedlings (yellow arrow in **I**; white arrows point to phloem files in the pedicel of cotyledon, which was removed, red arrows to leaf primordia). In a fully developed root tip, SYT4 partially colocalizes with PIN4 and is abundant mainly in columella initials and the outermost RC layer (**J**; white arrow points to PP, yellow to QC, blue to columella initials, purple to endodermis/cortex initials and red to lateral RC/epidermis initials, asterisk marks the outermost RC layer). **(K**, **L)** represent transversal sections of the root at different distances from QC and show fusion protein in developing phloem. The red signal is due to propidium iodide staining. SYT4 is abundant in PP (white arrows in **K**). More proximally, SYT4 is present in MP (yellow arrow in **L**), CC (red arrows), and less abundantly in PP (white arrow) and phloem PC (blue arrow; white asterisks mark protoxylem, yellow metaxylem and blue primary xylem elements). SYT4 accumulates at the periphery of developing PP **(M)** and SE **(N)**. In the inflorescence stem in the stage of primary growth, SYT4 is present exclusively in the phloem pole of PC (white arrows in **O;** red arrows point to the xylem poles of PC). Size bars – **(A, C–E, I–N)** =10 µm; **(F–H)** = 50 µm; **(B)** = 100 µm; **(O)** = 200 µm.

To examine the SYT4 protein expression pattern at the cellular and intracellular level, we created homozygous transgenic plants expressing SYT4-EGFP and SYT4-Dendra2 fusions under the native *SYT4* promoter and containing the endogenous 3′UTR sequence. In ovules and seeds, the fluorescence signal was detected only in the phloem at the chalazal end and funicules (**Figures 2A, B**). In developing embryos, fusion proteins first appeared in the shoot and root apical meristem cells at the late heart stage (**Figures 2C–E**) and in the PC tissue at the torpedo stage (**Figure 2F**). No signal was detected in fully developed dried seeds. A slight signal appeared in germinating embryos 24 hours after plating in the PC of hypocotyls and roots. After 48–72 h, the fusion protein was visible in immature PP elements of whole seedlings (**Figures 2G, H**) and detectable in the cells of shoot (**Figure 2I**) and root stem cell niche (**Figure 2J** and **Supplementary Figure 2B**). Here, the protein was most abundant in the columella initials but was also present in the quiescent center cells (QC), endodermis/cortex and lateral RC/epidermis initials, and the outermost layer of the RC but not in initials in the stele. Then, we examined the SYT4 patterning during phloem development using cross-sections. In the distal part of the root tip, only PP elements showed a fluorescent signal (**Figure 2K**). More proximally to the shoot, the protein also appeared in phloem PC and MP and on their sides localized CC (**Figure 2L**). In young developing sieves, SYT4 became concentrated as patches at the cell peripheries (**Figure 2M, N**). When we analyzed a 1 cm long inflorescence stem (the principal growth stage 5, inflorescence emergence; Boyes et al., 2001), the stem was in the stage of primary growth, and we detected a strong signal in the phloem pole of PC (**Figure 2O**).

### 3.6 Localization and dynamics of SYT4 in developing protophloem

**Figure 3:**
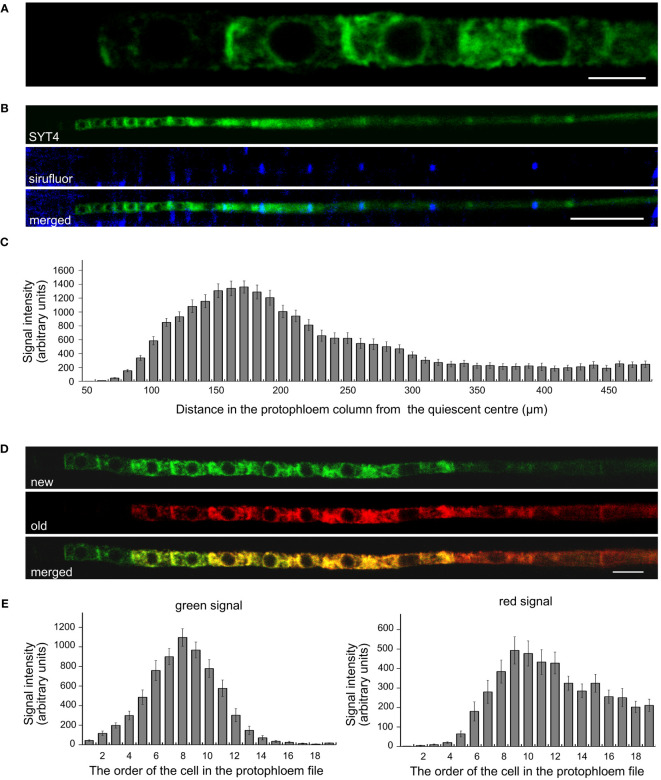
Localisation and dynamics of SYT4 in protophloem cells. **(A)** shows SYT4-GFP in first cells in the early dividing PP approximately 50 µM from the QC. SYT4 gradually disappears from PP cells, which deposit callose (blue signal due to sirofluor treatment) in their transversal cell walls (**B**; **C** shows signal intensities). **(D, E)** show the dynamic of SYT4 protein in PP estimated by SYT4-Dendra2 fusion protein. The decrease in SYT4 abundance in elongating PP cells is due to the discontinuing synthesis of the fusion protein (green signal in **D**) in differentiating PP cells. **(E)** shows values of the green signal emitted by the SYT4-Dendra2 population, which was synthesized within 4 h since photoconversion and red signal intensities emitted by the old photoconverted population. Size bars – **(A)** = 5 µm; **(B)** = 50 µm; **(D)** = 10 µm.

Precise cellular localization of SYT4 and its dynamics we studied in PP cells. Since the PP in the Arabidopsis root tip consists of only two columns of cells, these are readily observable, and the signal intensities of EGFP and Dendra2 are easily quantifiable with a confocal laser scanning microscope. We detected a slight signal already in the first early dividing PP cell approximately 50 µm from QC (**Figure 3A**, see also **Figures 2J, K**). However, incipient MP and PC file cells were negative within this distance from the QC. The signal was visible in PP meristematic cells as a network in the cytoplasm and a ring around the nuclei. It was also significantly enriched at the site of cell contacts of neighboring cells in the PP file (**Figure 3A**). The signal intensity increased dramatically in the more proximally located PP cells. However, as cells started to elongate and deposit callose in their transverse cell walls, the amount of SYT4 gradually decreased in the cells (**Figures 3B, C**). Using the SYT4-Dendra2 transgenic line, we demonstrated that the high abundance of SYT4 was due to its extensive synthesis in meristematic PP cells, as shown by the increasing intensity of the green signal emitted by the SYT4-Dendra2 population synthesized after the photoconversion. However, protein synthesis dropped in 11 to 14 cells in the PP column (**Figures 3D, E**). The abundance of the old red population of fusion protein synthesized before photoconversion was steadily reduced in differentiating SE.

### 3.7 SYT4 protein in lateral roots and secondary thickened organs

**Figure 4:**
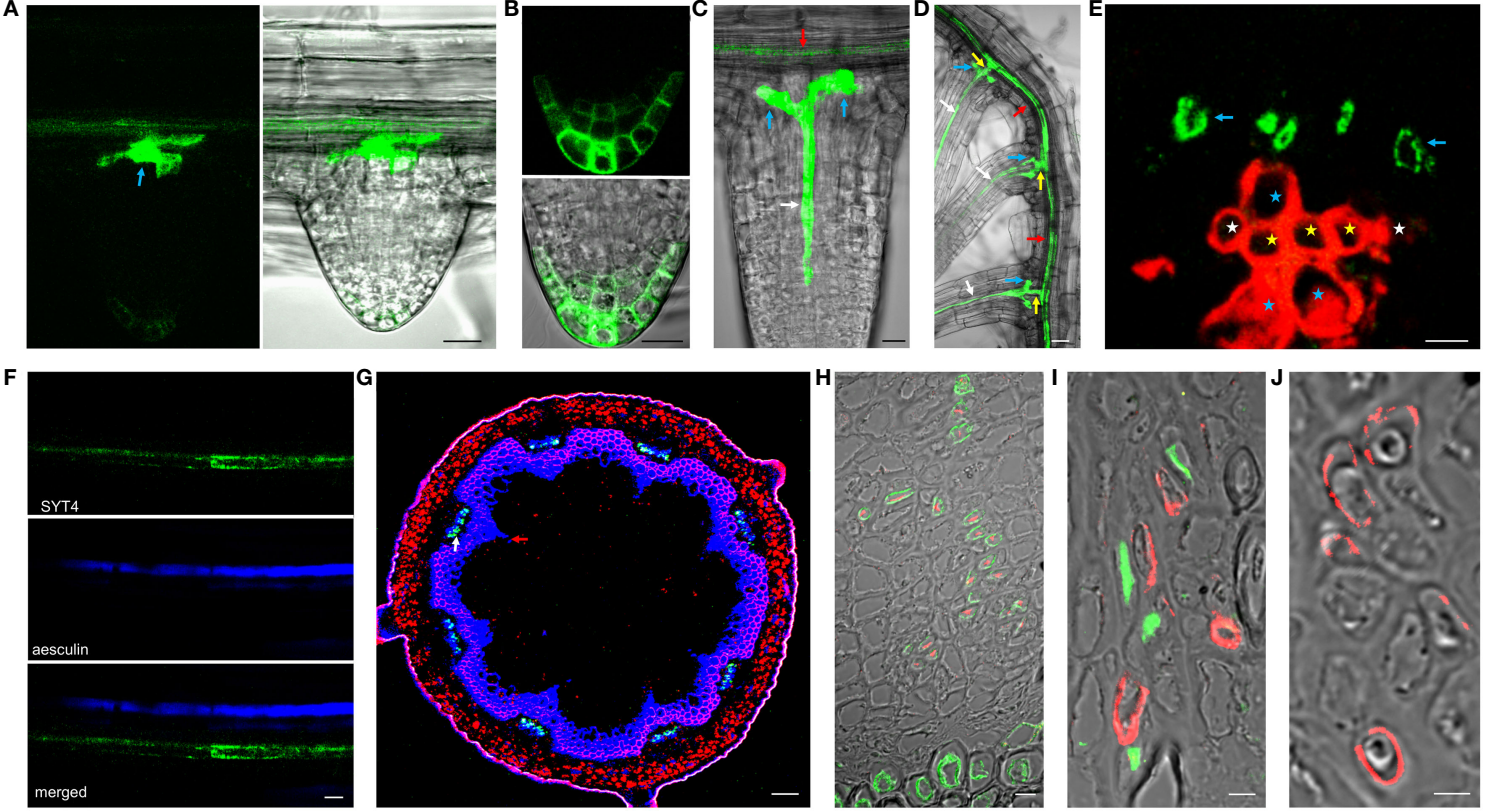
SYT4 in lateral root and inflorescence stem and primary root in the stage of secondary growth. SYT4 protein starts to be visible in emerging lateral roots. A strong signal is detectable in a group of cells in basal parts of roots (blue arrow in **A**) and also in the developing RC **(B)**, later in PP (white arrow in **C**) which grows up from the group of cells mentioned above (blue arrows; red arrow point to the phloem of the parental root) and finally also in the cells connecting the phloem of the lateral and the parental root (yellow arrows in **D**; arrows of other colors are as in **A**, **C**). In differentiated roots, SYT4 is present in phloem PC cells developing to primary SEs (blue arrows in **E;** white asterisks mark protoxylem, yellow metaxylem and blue xylem elements derived from PC) but not in fully developed sieve tubes accumulating aesculin (blue signal in **F**). In the inflorescence stem in the stage of secondary growth, the green signal is abundant in cells centripetally localized in the phloem (**G**; the blue signal is due to aniline blue staining; red fluorescence is emitted by chlorophyll). **(H–J)** show histoimmunological treatment of transverse sections of the primary roots in the stage of secondary growth. SYT4-Dendra2 protein labeled with the antibody against Dendra2 (red signal) is visible in cells labeled with JIM13 specific antibody (green signal in **H**) but not in cells labeled with the LM5 antibody (green signal in **I**). In this case, the inner phloem layers’ cells labeled with the Dendra2 antibody (green signal) are located close to cells labeled with LM5 antibody **(I)**. In old phloem regions at the root periphery, Dendra2 labeling is absent in cells adjacent to cells reacting with LM5 antibody **(J)**. Size bars – **(I, J)** = 5 µm; **(E, F, H)** = 10 µm; **(A–C)** = 20 µm; **(D)** = 50 µm; **(G)** = 100 µm.

We could not detect the signal in early lateral root primordia (**Supplementary Figure 2D**). However, the intense fluorescence appeared firstly in emerging lateral roots from parent roots in a group of cells in the central basal domain, and the weaker signal was seen in the central apical domain (**Figure 4A**). Here, SYT4 became visible during new root stem cell niche establishment (**Figure 4B**). Later, the signal was strong in the PP files, connected with the group of cells mentioned above (**Figure 4C**), and finally, in the cells connecting the SE of lateral and primary roots (**Figure 4D**). As described above, SYT4 was abundant in SE precursors and less profuse in maturing elements. We wanted to verify this finding by studying more developed organs. On the cross-section of differentiated root parts, cells exhibiting green fluorescence were scattered in phloem poles but were no longer present in the PP/MP areas (**Figure 4E**). We also observed the signal in several parallel cell files in the phloem; however, rarely in functional sieve tubes labeled by aesculin (**Figure 4F**). Then, we analyzed shoots and roots undergoing secondary growth. On the free-hand sections and cryosections, we saw SYT4 only in centripetally located cells of the phloem. Here, the green signal partially overlapped with the blue signal emitted by aniline-stained callose deposited in maturing sieves (**Figure 4G**). Centrifugally located cells showed only a blue signal. SYT4 distribution contrasted with the appearance of SYT1 protein, which was present in different tissues (**Supplementary Figure 2I**). Also, in the phloem, SYT1 was visible in all cell types (**Supplementary Figure 2J**), whereas SYT4 was accumulated at the periphery of particular phloem cells (**Supplementary Figure 2K**).

Since cells in the phloem of Arabidopsis organs are tiny and difficult to specify without fixation and proper sectioning, we employed histoimmunology techniques to identify cells expressing SYT4. We co-labeled sections of roots with the Dendra2-specific antibody and JIM13-specific antibody recognizing SE (Šamaj et al., 1998) and LM5-specific antibody recognizing CC (Torode et al., 2018), respectively. The signal for Dendra2 was localized within cells, with the cell walls labeled with JIM13 (**Figure 4H**). When we combined antibodies against LM5 and Dendra2, labeling was detected in distinct but adjacent cells in phloem islets in the inner part of the phloem (**Figure 4I**). However, in outside phloem layers, labeling of Dendra2 was not detected (**Figure 4J**).

### 3.8 Isolation and analysis of *syt4* T-DNA insertion alleles

**Figure 5:**
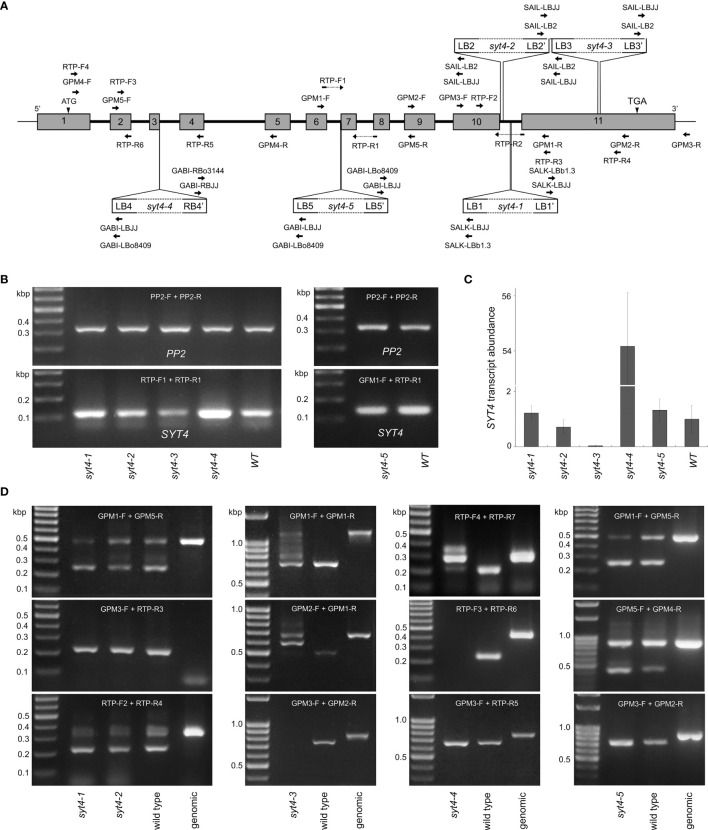
*SYT4* transcripts in mutant alleles. **(A)** indicates the locations of T-DNA inserts and positions of primers employed in genotyping (**Supplementary Figure 1**) and qPCR. **(B)** proves the occurrence of *SYT4* transcript in mutant alleles by semi-quantitative RT PCR using primers overlapping introns 6 and 7 (**A**; run for 35 cycles). The same primers were used for real-time qPCR (**C**; transcript abundance is expressed as a multiple of the average abundance in the wild type, n=3). **(D)** shows RT-PCR products in *syt4* alleles obtained by different combinations of primers (their positions are given in **A**) by extended numbers of cycles (40–45).

Based on available data from the TAIR database, we selected five mutant alleles of *SYT4* for deep analysis. By standard procedures, we isolated two homozygous lines for each selected allele. By PCR-based approach, we showed the presence of inserts and the absence of the entire *SYT4* gene in all lines. The positions of primers are in **Figure 5A**, and the results of PCR genotyping are in **Supplementary Figure 1**. Then, we re-evaluated the positions of T-DNA insertions by sequencing PCR fragments. We have established that four alleles, *syt4–1*, *syt4–2*, *syt4–3* and *syt4–5* contained complex T-DNA inserts. The inserts occurred as inverted T-DNA repeats around the right border, so they are bound to the *SYT4* gene loci by left borders. The *syt4–4* contains a single T-DNA molecule or direct T-DNA repeats with both LB and RB at junctions. Next, we verified the T-DNA insertion sites in the genomes and completed the missing T-DNA/plant DNA junction sequences for all mutant alleles. Flanking insertion sequences from the TAIR database indicated that the T-DNA insertion disrupted exon 10 in the Salk *syt4–1*; however, we identified that this allele had inverted T-DNA repeats in the 10th intron at 2144 bp downstream of the start codon. Also, the Syngenta *syt4–2* allele with expected T-DNA insertion in the exon 10 had this insert actually in the 10th intron at 2193 bp downstream of the start codon and 23 bp were deleted during the integration process. As expected, the *syt4–3* allele had integrated T-DNA insert in exon 11, precisely at 2637 bp downstream of the start codon and 10 bp were deleted. According to the original data, the *syt4–4* GABI-Kat mutant should have integrated T-DNA in the second exon; however, we have found that this position was actually in the third intron at 448 bp downstream of ATG. Finally, in the *syt4–5* GABI-Kat allele, the T-DNA was linked to the genome by one of the left borders in the intron 6 as previously determined, precisely at 1346 bp downstream of the start codon; however, the second LB of inverted repeat was attached to the genomic sequence at the beginning of exon 7 and 15 nucleotides were lost from the genomic sequence. The junction sequences, structures of T-DNA insertions and consequences caused by insertions in the mutant alleles are given in **Supplementary Table 3**.

### 3.9 *SYT4* transcripts in mutant alleles

We then tested the presence and patterns of *SYT4* transcripts in all *syt4* alleles by RT-PCR. By qPCR we quantify the abundance of *SYT4* transcript combining primers designed to bridge exon 6/7 and exon 7/8 junctions (**Figure 5A**) to anneal specifically to *SYT4* cDNA. For the *syt4–5* allele, we used a forward primer located slightly more upstream since the original forward primer partially overlapped the deleted DNA stretch. This procedure with primers that anneal in the middle of cDNA and PP2A as reference gene revealed more than 50 times increased abundance of *SYT4* transcript in *syt4–4* roots and almost 30 times decreased abundance of the transcript in *syt4–*3 roots, while in *syt4–1*, *syt4–2* and *syt4–5* transcript abundance was affected only slightly (**Figure 5C**). The pattern obtained in semi quantitative RT-PCRs with the same primers and 30 cycles shown in **Figure 5B** confirmed the synthesis of fragments of the correct size for *SYT4* cDNA and the absence of genomic contamination.

Further, we tested all mutant alleles with different combinations of primers and an extended number of cycles (40–45) to see how *SYT4* transcript look in different *syt4* alleles. Positions of primers are accessible in **Figure 5A**. In alleles *syt4–1* and *syt4–2*, which have insertions in the 10th intron, all combinations of primers from upstream of insertion sites produced fragments of the correct size for *SYT4* cDNA. An example is presented in **Figure 5D**. If we employed the reverse primer, which bridges the exon 10/11 junction in combination with the forward primer positioned in the exon 10, upstream insertion site, we obtained the same fragment as in the wild type, and the fragment size corresponds to cDNA suggesting splicing in this site was not affected by T-DNA inserts in the intron 10. Genomic DNA does not produce the amplicon. When we analyzed the *syt4–3* allele, which has an insertion in the exon 11, we obtained fragments with all primer pair combinations from upstream of the T-DNA insert. The patterns were not identical to those of wild-type plants because multiple amplicons were produced in the *syt4–3* allele. An example is in **Figure 5D**. When we combined primers from both sides of the T-DNA insertion, we failed to amplify the fragment in mutant plants (**Figure 5D**). In the *syt4–4* allele, which contained T-DNA in the third intron, RT-PCR fragments were obtained using primers annealing upstream or downstream of the insertion site; however, primer combinations from both sides of the T-DNA insert have not produced fragments (**Figure 5D**). In the *syt4–5* allele, which has the T-DNA insertion at the intron 6/exon 7 border, all combinations of primers produced fragments of the correct size for *SYT4* cDNA, similar to the wild type (**Figure 5D**).

### 3.10 Phenotype analysis of *syt4* mutants

**Figure 6:**
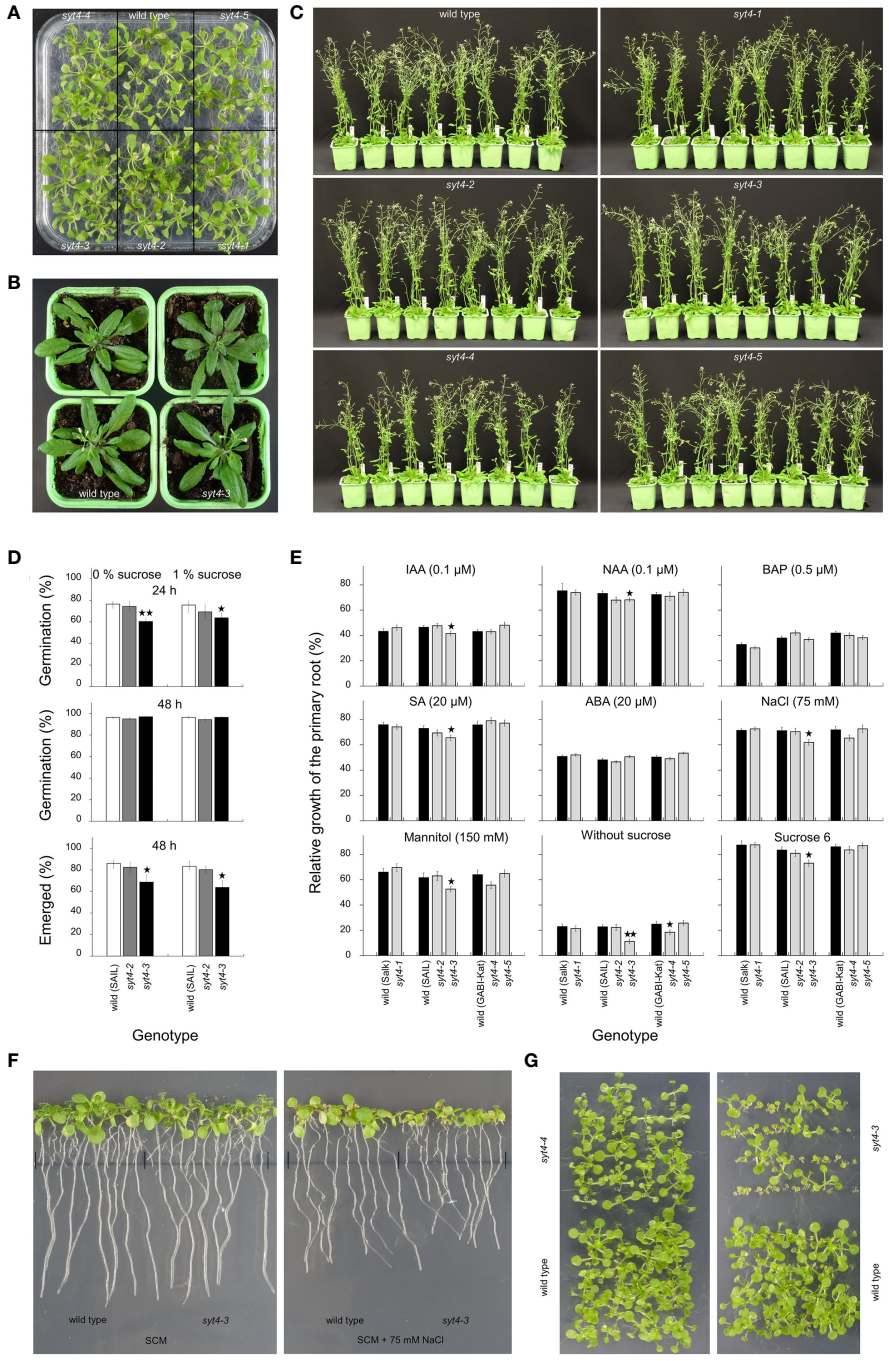
Phenotype analysis of *syt4* alleles. **(A)** shows seedlings germinated and grown on SCM with 1% sucrose. Two wild-type plants and *syt4–3* alleles in the stage of inflorescence emerging/the first flower open are shown in **(B)**. **(C)** shows wild-type plants and *syt4* mutants in the growth stage 6.50 (Boyes et al., 2001). Differences between the wild type, and, *syt4–2* and *syt4–3* alleles in germination rates and proportions of seedlings with fully emerged hypocotyls and cotyledons from the seed coat are shown in **(D)** (differences between the wild type and mutant alleles are indicated by single asterisks for p ≤ 0.05 and double asterisks for p ≤0.01, n=6, together more than 1000 seeds were analyzed for each genotype). Influence of different factors on elongation of primary roots of *syt4* alleles and corresponding wild types are accessible in **(E)** (differences between the wild type and mutant alleles are indicated by single asterisk for p < 0.05 and double asterisks for p < 0.01, n ≥80). **(F)** demonstrates the difference in the growth of primary roots between the wild type and *syt4–3* allele when grown for the last three days on SCM supplemented with 75 mM NaCl. In **(G)**, seedlings of *syt4–3*, *syt4–4* mutants and wild type germinated and grew on SCM without sucrose.

Firstly, we looked at the appearance of *syt4* mutant plants growing in *in vitro* conditions on SCM supplemented with 1% sucrose under a 14 h light at an intensity of 150 µmol m^-2^ s^-1^/10 h dark photoperiod. We did not observe changes in growth in any of the five alleles, but they resembled the wild type (**Figure 6A**). Then, we estimated plants growing in pots. We did not observe differences in phenotype between mutants and corresponding controls (*syt4–3* mutant and control plants in the stage of fluorescence emergence are shown in **Figure 6B**). Then, we compared more advanced plants (growth stage 6.50 as characterized by Boyes et al., 2001) and failed to find growth defects in insertional alleles (**Figure 6C**). We checked siliques and observed normal seed development in all mutant alleles.

Then, we estimated if a mutation in the *SYT4* gene affects seed germination. When we analyzed the germination rate after 48 h, we failed to find differences between the wild type and two *syt4* alleles (**Figure 6D**). However, observation after 24 h showed that the *syt4–3* allele, but not the *syt4–2* allele which show no altered transcript, is delayed in germination compared to the wild type (**Figure 6D**). This was confirmed by observing the proportion of fully emerged seedlings from seed coat after 48 h (**Figure 6D**), which was significantly lower in *syt4–3* alleles than in the wild type. The presence or absence of sucrose did not play a role in these processes. Finally, we performed the primary root growth test. We took advantage of the knowledge from studying the *SYT4* promoter and estimated the influence of factors affecting its activity to find a possible effect on the mutants’ phenotype. The growth increment of roots of *syt4–1*, *syt4–2*, and *syt4–5* alleles was not different from wild types under any of the investigated factors (**Figure 6E**). When we analyzed the *syt4–4* mutant, we observed a slight relative decrease in root elongation under conditions such as mannitol and NaCl; however, a statistically significant difference between the wild type and *syt4–4* was detected only under the absence of sucrose (**Figure 6E**). The root growth in the *syt4–3* mutant was significantly more reduced than in the wild type under the influence of all factors except BAP and ABA, and the difference was highly significant when seedlings were grown without exogenous sucrose (**Figure 6E**). The influence of sodium chloride is shown in **Figure 6F**. The importance of exogenous sucrose for the growth of *syt4–3* and *syt4–4* mutants is also confirmed by extended cultivation of seedlings *in vitro*. An increased number of stunted seedlings was apparent in both cases, and in the *syt4–3* allele, the number of such seedlings was higher than in the *syt4–4* allele (**Figure 6G**).

## 4 Discussion

Plant SYTs resemble animal SYTs and E-SYTs in structure. The ubiquitously and most highly expressed *SYT1* in Arabidopsis has been studied extensively, but the role of other members needs to be better understood (Benitez-Fuente and Botella, 2023 for review). Recent studies propose the role of SYT1 in synergy with other SYTs at ER/PM contact sites (Pérez-Sancho et al., 2015; Siao et al., 2016; Ruiz-Lopez et al., 2021), similar to E-SYTs in animals (Nath et al., 2020). In this study, we characterize *SYT4*, which has not yet been described. This gene, like *SYT1*, is also generally expressed in all organs, as we show here by analyzing *SYT4* promoter strength and the abundance of the *SYT4* transcript. However, detailed analysis showed that, in fact, in all organs, *SYT4* is expressed, with few exceptions, only in the phloem tissue, which is not the case of *SYT1.* The specific expression of *SYT4* is not particularly surprising, as, for example, the *SYT2* promoter is active only in female and male gametophytes, and *syt2* is defective in pollen germination and pollen tube growth (Wang et al., 2015). On the other hand, *SYT1*, which is highly homologous to *SYT2* (García-Hernández et al., 2024), is constitutively expressed across most tissue types, including phloem (Schapire et al., 2008, **Supplementary Figures 2I, J**). However, protein localization patterns of SYT4 and SYT1 in phloem are not identical (compare **Supplementary Figures 2J, K**). The functional diversity of SYTs in animals is also well-documented (Wolfes and Dean, 2020).

The pattern of *SYT4* promoter activity is very similar to that seen in promoter PD2 or PD5 early phloem differentiation marker lines (Bauby et al., 2007). Similarly, *SYT4* promoter activity was detected already in PP, as shown in this study. When we studied fusion protein expression, this was in our transgenic lines evident even in younger PP cells than in the PD2 and PD5 marker lines, i.e., 50–60 micrometers from RC junction (**Figure 2J**). At this distance, PP and MP files are formed from a common precursor (Blob et al., 2018), so SYT4 appears in the first cell in the PP strand. Only a few genes are known to be expressed explicitly in the first cells of PP, i.e., *NAC DOMAIN-CONTAINING PROTEIN 20 (NAC020)* or *ALTERED PHLOEM (APL)* expressing slightly more proximally (see Blob et al., 2018 for review). Both genes encode MYB transcription factors, important regulators of SE differentiation. Therefore, our transcription and transcription-translation *SYT4* transgenic lines can be good tools for studying the development of SE in different organs. The development of SE in the lateral root primordia is an excellent example. We recognized a separate group of cells in the basal central domain of lateral root primordia strongly expressing *SYT4* (**Figure 4A**). These cells seem to serve as a starting point for the differentiation of PP and elements connecting SE of lateral roots with parental roots (**Figures 4C, D**). In general, the process of differentiation of these connections is poorly understood. According to Torres-Martínez et al. (2020) phloem pericycle pole cells are capable of becoming founder cells and progeny of these cells could participate in the establishing connection between the parent and lateral roots to maintain continuity of phloem elements. Precise identification of cells in the basal central domain of lateral root primordia expressing *SYT4* requires deeper studies using specific markers.

The developmental trajectory of SE is unique in plants and includes the speedy degradation of cellular structures, including the nucleus (Hardtke, 2023). By photoconvertible Dendra2 tag, which was involved in research in our previous studies (Jásik et al., 2013; Jásik and Schmelzer, 2014; Jásik et al., 2016; Lešková et al., 2019), we demonstrated that synthesis and accumulation of SYT4 protein, at least in PP, are maximal in cells that start to differentiate into SE. The massive occurrence of SYT4 in SE precursors suggests that protein might play a significant role in the autophagous digestion of intercellular content. Interestingly, in HeLa cells, ER/PM contact sites are essential for autophagosome biogenesis; furthermore, early autophagic markers are recruited to E-SYTs-containing domains during autophagy, and finally, inhibition of E-SYTs expression leads to a reduction in autophagosome biogenesis (Nascimbeni et al., 2017). The second possibility is that SYT4 behaves similarly to classical SYTs in the secretion. The cell wall of differentiating SE thickens and deposits callose. Exocytosis plays an essential role in these processes. However, on the other hand, the expression of SYT4 in the embryo PC could suggest that this protein might already play a role in the establishing and development of this meristem.

Interestingly, in the root region where PP undergoes enucleation, *SYT4* is also expressed in surrounding cells, i.e., CC, MP, and phloem pole pericycle cells (**Figures 1B, C, E**, **2L**). This situation is unique for this stage because in older roots and stems, SYT4 is present only in developing SE and not in CC, as we have shown in the root by immunohistochemistry using an LM5-specific antibody (**Figures 4H–J**). Recently, several genes showing a ‘ring’ pattern, i.e., expressed in cells around enucleating PP, were identified (Otero et al., 2022). Most of them are transcription factors, including APL, mentioned above. This MYB transcription factor is expressed in PP and, at the time of enucleation, is transcriptionally activated in surrounding cells. Remarkably, there are several MYB cis-elements in the *SYT4* promoter (**Supplementary Table 2**). Perhaps *SYT4* expression is activated by this transcription factor.

Furthemore, SYT4 protein is abundant in the root tip, specifically in several cell types in the root stem cell niche and the outermost layer of the RC (**Figure 2J**, **Supplementary Figure 2B**). Interestingly, other marker lines for PP development, such as PD2 and PD3, also show expression in the differentiating RC (Bauby et al., 2007). RC cells undergo programmed cell death and produce many secretes released by secretory vesicles to the cell surface (Kumpf and Nowack, 2015; Driouich et al., 2021). So again, SYT4 may act as E-SYT or classical SYT in these cells. On the other hand, SYT4 is also abundant in the root stem cell niche, namely QC, and columella, endodermis/cortex and epidermis/lateral RC initials. The occurrence of protein already in developing embryos suggests a significant function in differentiating its root tip and shoot apical meristem. Notably, cells expressing SYT4 in the root tip are not developmentally related to the phloem. In fact, we have never observed SYT4 in vasculature initials of the root stem cell niche, which give rise to PP precursor cells among other tissues. It is well known that the proper root stem cell niche function requires a reciprocal and bilateral exchange of signals between the QC and surrounding stem cells (Pardal and Heidstra, 2021). Several molecular factors that regulate root stem cell homeostasis that either promote or inhibit QC division have been identified (Dubrovsky and Vissenberg, 2021; Strotmann and Stahl, 2021). Among them, transcription factors, WUSCHEL-RELATED HOMEOBOX 5, PLETHORA, SCARECROW, and SHORT-ROOT, the signal peptide CLAVATA3, or various plant growth regulator-responsive factors have been intensively studied. However, the connection of plant SYTs to networks of these proteins needs to be clarified. In general, there are only a few interactors of plant SYTs, such as aquaporins, two ubiquitin-related proteins, the SUMO-conjugating enzyme SCE1, one ammonium transporter and syntaxin-23, listed in the Biogrid database (https://thebiogrid.org/1986/summary/arabidopsis-thaliana), in contrast to animal SYTs and E-SYTs, where dozens of interaction partners are known.

To show the possible function of the *SYT4* gene in phloem and root tip development, we, after comprehensive transcript analysis, estimated the phenotype of five T-DNA insertional mutant lines. For all alleles, we did not observe apparent overall abnormalities in their phenotype when the plants were grown *in vitro* on SCM with 1% sucrose or pots under standard conditions (**Figures 6A–C**). Interestingly, the same situation was for the *syt1–2* allele (Schapire et al., 2008), which is regularly employed to study the *SYT1* gene function. We hypothesized that, as in this case, the phenotype of *syt4* mutants could appear *in vitro* under specific conditions. We took advantage of the knowledge gained from studying *SYT4* promoter. In fact, we found several factors, such as sucrose, ABA, mannitol, auxins, and NaCl, to affect *SYT4* promoter activity (**Figure 1R**). These results agree with the presence of cis-elements in the *SYT4* promoter (**Supplementary Table 2**). The failure to find a phenotype in any case for the three alleles, i.e., *syt4–1, syt4–2* and *syt4–5*, is not a surprise as RT-PCR and qPCR analyses showed that the T-DNA insertions did not alter transcript appearance and abundances in these alleles.

In *syt4–4* allele, the primary root growth test showed significant inhibition of root elongation only in the absence of sucrose. The *syt4–3* allele, in which, according to RT and qPCR analysis, the *SYT4* transcript is altered at 3´end and much less abundant than in wild-type plants, showed a significant effect of several factors such as auxins IAA, 2,4-D, SA, mannitol, NaCl, or the absence or excess of sucrose in the medium. We also observed a delay in germination in this mutant. Of particular note is the inhibition of root growth in the absence of exogenous sucrose. The lack of sucrose was also reflected in the growth inhibition of *syt4–3* seedlings under *in vitro* conditions (**Figure 6G**). One could speculate that the retarded growth of the seedlings and the mentioned root elongation could be related to the altered phloem function due to the absence of the *SYT4* gene function. Thus, the long-distance transport of sucrose between the source (leaves of seedlings as the only source in the lack of exogenous sucrose in the medium) and the sink (roots), which is mediated by phloem, could be disturbed. However, why a similar effect does not occur in the case of *syt4–3* plants growing in soil is unclear.

Given the particular expression of the *SYT4* gene in the phloem, even in PC and apical meristems of embryos at early stage of development, one would expect marked changes in the phenotype in the absence of *SYT4* gene function. Usually, phloem or apical meristem-specific gene mutations cause significant alterations in root growth and development (Blob et al., 2018; Dubrovsky and Vissenberg, 2021; Pardal and Heidstra, 2021; Strotmann and Stahl, 2021). One possible explanation is that the available T-DNA insertion *syt4* alleles are actually not null mutants, and *SYT4* may be fully or partially functional in all five insertion alleles. It should be noted that *syt4* alleles have not been characterized. Only the *syt4–4* allele was published to affect resistance to *Pseudomonas* (Kim et al., 2022) but not subjected to molecular biology analysis. First of all, the insertion sites in the TAIR database for the *syt4–1, syt4–2* and *syt4–4* alleles were imprecise, as we demonstrate in this study. In fact, the T-DNAs were incorporated in introns and not in exons, as previously reported. Allele *syt4–5* has insertion in intron as expected but we have shown that second junction site is in exon. Despite this, the full transcript was present, probably due to alternative splicing. Anyway, the allele never shows altered phenotype. Only the *syt4–3* allele has inserted T-DNA in the last exon, as predicted. However, even in this case, the incorporation was relatively close to the stop codon. Generally, T-DNA insertions in introns eliminate gene function less likely than those in exons (Wang, 2008), as insertions in introns do not necessarily disrupt normal transcript splicing. According to our findings, this is true for *syt4–1* and *syt4–2* alleles with T-DNA inserts in the intron 10 and the *syt4–5* allele with the insert in the position intron 6/exon 7. All three alleles produce complete transcripts. qPCR showed only a slight variability in transcript abundance in all three alleles when the pair of primers binding specifically to *SYT4* cDNA in its middle part were employed (**Figures 5B, C**). We also found no effect of T-DNA insertion on transcription with several combinations of primers (**Figures 5D, G**). On the other hand, the insertion in the last exon apparently disrupted the splicing process in the *syt4–3* allele (**Figure 5E**). Interestingly, according to a transcriptomic study on Arabidopsis roots (Huang et al., 2022), *SYT4* produces multiple splicing forms; however, their relative abundance in different organs has yet to be analyzed, and the function of alternative splicing has yet to be defined for this gene. Finally, qPCR shows dramatically decreasing transcript abundance in this allele when analyzed with primer pair binding in the middle region of cDNA (**Figure 5C**). In the case of *syt4–4*, which has the T-DNA insert in intron three, we identified amplicons with all primer pairs binding to cDNA downstream of T-DNA insertion (**Figure 5F**). qPCR revealed significantly increased transcript abundance compared with wild-type plants, which is highly probable due to the 35S promoter at the right border of the T-DNA. Another explanation for the lack of a pronounced phenotype in *syt4* alleles is that *SYT1*, the most highly expressed gene in the Arabidopsis *SYT* family (Schapire et al., 2008), and *SYT4* have redundant functions. Such scenarios have been proposed for SYT3 (Ruiz-Lopez et al., 2021) and SYT5 (Lee et al., 2020) regarding their possible substitutable roles with SYT1 at ER/PM contact sites in leaf and cotyledon epidermal cells. Indeed, the *SYT1* promoter is highly active in all tissues but markedly so in vascular bundles (Schapire et al., 2008). We analyzed the distribution of SYT1 and found that this protein is highly abundant in phloem cells but not restricted only to developing SE and their precursors, as is the case of SYT4 (compare **Supplementary Figures 2J, K**).

In conclusion, SYT4, a hitherto undescribed gene of the Arabidopsis synaptotagmin family, is specifically expressed in the phloem, particularly in SE precursors and developing sieve tubes and some types of stem cells in apical meristems. Thus, our transcription and transcription-translation *SYT4* transgenic lines can be good tools for studying the development of SE in different organs. Expression pattern implicates a role for SYT4 in SE development/function and apical meristem organization. More information on the role of the gene could be conveyed by the phenotype analysis of the *syt4* mutants; however, our findings showed that the insertion alleles for this gene available in publicly available collections are not completely knock-out alleles. We analyzed up to 5 lines, and only the *syt4–3* allele , which has C2B domain interrupted by the T-DNA insertion showed consistently significant delayed germination and suppressed root elongation. This has also been only observed in *in vitro* conditions under various factors, especially in the absence of exogenous sucrose. Interestingly, the *syt1–2* allele has an insertion at a similar site in the C2B domain and also exhibits a phenotype only under the influence of sodium chloride stress. It is quite unusual that the phenotype in the mutants of the highly and ubiquitously expressed *SYT1* and very specifically expressed *SYT4* have no apparent phenotypes in normal conditions. The genes may have a redundant function; therefore, constructing double *syt1/syt4* mutant lines and subsequent phenotyping could provide more information about the functions of both genes. Even better would be to establish proper knock-out lines for *SYT4* using state-of-the-art methods such as the CRISPR/Cas9 approach.

## Data availability statement

The raw data supporting the conclusions of this article will be made available by the authors, without undue reservation.

## Author contributions

AK: Investigation, Methodology, Formal analysis, Writing – original draft, Writing – review & editing, Validation, Visualization. MK: Methodology, Writing – review & editing. JJ: Conceptualization, Formal analysis, Funding acquisition, Project administration, Supervision, Writing – original draft, Writing – review & editing, Investigation, Resources.

## Supplementary material

Supplementary Figure 1 Confirmation of the presence of T-DNA inserts and homozygosity of *syt4* alleles.

Supplementary Figure 2 Analysis of the *SYT4* promoter activity and SYT4 and SYT1 protein distribution. The root with the strong *SYT4* promoter activity in the RC is in **(A)**. **(B)** shows the distribution of SYT4 protein in the root stem cell niche (red asterisk marks columella initials, purple QC, cyan stele initials, red arrows point to epidermis/lateral root cap initials, purple to endodermis/cortex initials and yellow to pericycle initials). **(C)** shows the activity of the *SYT4* promoter and **(D)** SYT4 protein pattern in the root region with a lateral root primordium (arrows). **(E)** shows *SYT4* promotor activity in root tips of 10 days seedling growing on SCM. In **(F)**, a seedling was grown for the last 2 days on SCM supplemented with 1 µM 2,4-D, **(G)** with 300 mM mannitol and **(H)** with 150 mM NaCl. The section through the shoot of the *SYT1-Dendra2* plant documenting the occurrence of SYT1 protein in all tissues is shown in **(I)** (white asterisk marks cortex, red phloem, blue cambium, yellow xylem, violet pith). The close view of the phloem region is in **(J)**. **(K)** shows the phloem region of the *SYT4-GFP* shoot (asterisks in **J** and **K** are as in **I**). Size bars – **(A–D, I–K)** = 20 µm; **(E–H)** = 100 µm.
