# Supplementary material for: SYNAPTOTAGMIN 4 is expressed mainly in the phloem and participates in abiotic stress tolerance in Arabidopsis
Source: Front Plant Sci. 2024 Jul 1;15:1363555. doi: 10.3389/fpls.2024.1363555 (PMC11246894; doi:10.3389/fpls.2024.1363555)
Supplement: Supplementary file 1 [file Table_1.docx]

**Supplementary Table 1**: List of primers

| **Purpose** | **Primer names** | **Sequences (5’-3’)** |
| --- | --- | --- |
| **Genotyping and RT-PCR**  **of syt1-2** | RTF1 | TAGTTCCAATCCTTGACCCTGC |
|  | RTR1 | CTCTAGAACCTGAGTCTGGGGA |
|  | RTF2 | TCCCCAGACTCAGGTTCTAGAG |
|  | RTR2 | ATTTCTTCCTCCGTGAATGGCT |
|  | RTF3 | TGGACGTGGTGAACAACAAA |
|  | RTR3 | AGCTCGATTTGGATCTTTCCGT |
|  | RTF4 | AACGCTGATCAATTCCACAGTTTTC |
|  | RTR4 | GAAGGCTCTGCAAATCTGAATG |
|  | GF | GCTG CTGGAGGAAT GCTTGTGGTA ATAGTG |
|  | GR | TCAAGAGGCAGTTCGCCACTCGAG |
|  |  |  |
|  |  |  |
|  |  |  |
|  |  |  |
| **Genotyping and PCR amplicon sequencing** | GPM3-R | AAAAGAAGGTCACCCCTGAAG |
|  | GPM4-F | AGGAATCGCGGTTTCTTTCGGT |
|  | SALK-LBJJ | CCCTATCTCGGGCTATTCTTTTG |
|  | SALK-LBb1.3 | ATTTTGCCGATTTCGGAAC |
|  | SAIL-LBJJ | GCATCTGAATTTCATAACCAATCTCGATACA |
|  | SAIL-LB2 | GCTTCCTATTATATCTTCCCAAATTACCAATACA |
|  | GABI-LBJJ | ATAACGCTGCGGACATCTACA |
|  | GABI-LBo8409 | ATATTGACCATCATACTCATTGC |
|  | GABI-RBJJ | TCCAACCACGTCTTCAAAGCA |
|  | GABI-RBo3144 | GTGGATTGATGTGATATCTCC |
| **Genotyping and RT-PCR** | GPM1-F | CTTGCTTTGGGGCACTTTC |
|  | GPM1-R | CTTTCCGGCTTCAGTACCTTC |
|  | GPM2-F | TGTGAAGGTTGTGCAAGCAAAG |
|  | GPM2-R | ATGGACACAAAGCTTCCCTG |
|  | GPM3-F | AGTTCATTGTGGAAGATGTCTCAAC |
|  | GPM4-R | TCCATTGTGATTCCATTAGGTCCA |
|  | GPM5-F | AGACCATTGCTGCATTTGCAA |
|  | GPM5-R | TGCTTGCACAACCTTCACATCCA |
| **RT-PCR** | RTP-F1 | CTTATTCACTCCGCGAGAAGAAAG |
|  | RTP-F2 | ATTGGGGCTGCTCAAGTACC |
|  | RTP-F3 | TGACAGTTCAAGATTCGAGAAAACTT |
|  | RTP-F4 | CATCCATGGGTTTTCTCTTTGGTTTGTTCATAG |
|  | RTP-R1 | AATGGCGTCACGAATAGTTTCTTC |
|  | RTP-R2 | TATAAGAGCTCCAACTGCACCTGAC |
|  | RTP-R3 | GCTTTCCGGCTTCAGTACCT |
|  | RTP-R4 | TGACGCGAGTGATGTTAGAAGGAG |
|  | RTP-R5 | AGTCCCAAGTGTGAATTTAGAGAACT |
|  | RTP-R6 | TGTCTCTGAGAAAACACAACCCAAG |
| **RT-PCR of reference genes** | PP2A-F | AATCGTTACTGCCAGCCATTGTA |
|  | PP2A-R | AGAAGCGATACTGCACGAAGAAT |
|  | MON1-F | AAAGGATTGGGACCCCACAA |
|  | MON1-R | CTCTCAAGGGTTTCTCGGTACA |
|  |  |  |
|  |  |  |
