# Supplementary material for: SYNAPTOTAGMIN 4 is expressed mainly in the phloem and participates in abiotic stress tolerance in Arabidopsis
Source: Front Plant Sci. 2024 Jul 1;15:1363555. doi: 10.3389/fpls.2024.1363555 (PMC11246894; doi:10.3389/fpls.2024.1363555)
Supplement: Supplementary file 2 [file Table_2.docx]

**Supplementary Table 2:** List and position of cis-elements in *SYT4* promoter sequence

CTAGAAAGATTCTGTGTTTAAGAAATTTAAAATCATAAAGGGTGTTTTTTTCCTTATGCT -1981

TATGCGAATATAAGGGCTGTTATGAATTTCCAAGATTCGTTGGGACGCAAATTTATGCAT -1921

TTCACATTCATATATCGAGAAAAAAATTGGCCAAAAAGTAAGAGAAGTTCCTAGATTCGT -1861

TGGGACACAGATTTATGCATTTCACATTCATATCTCAAGGAAATTTTTTAAAGTAAGTCT -1801

AAAAATCATAAGCACAAAAGATTGTGTTCGAAGCATAAATTAAGTAGTACCATGAGTTTC -1741

CATCACATTCAATCTTAATCAATTTCAATGATGCTTTTCCTTTGTTCTTTCTCATTATTC -1681

ACGAATAGTGAAAATATAGGACAAAACATAACAATTTCATTGTTAATGCATGTATAATAA -1621

TCACAAAATCCCAATTATTAGTTTTGATAAATGACTA**CAACAG**TATTTTTAATTAGGTTT -1561

**MYB binding**

ATGTCTATCTAATTAACGCCTATAAATTTGGTTACAACATTATTGCTTTTTTTTGTCGAC -1501

AACATAGCTTTTTTCTTTAAACATATAAATGACAA**CACGTG**ATATATCAAATTCGGCCTA -1441

**G-box,ABRE**

ATACATAATG**TAACTG**ATGATGAAATTGATAAAGCTACTTGTTGAAACAGTATATTTACA -1381

**MYB biding**

CTCATTTCTAACGATACGTTACCTAATGC**TAACCA**AACAAAATTTCAATATGAATCGCGT -1321

**MYB recognition**

ACTTAGAA**CAACCA**TCTTATATTATGCATTGATTATATAATACTATTCAAAGTCAATGCT -1261

**MYB binding**

TTGTAGTATCTTTCGTTTCGTGGTAAGTTCAAATACTCATATTCAAGATAATACAATCGG -1201

GACCGGTCATAAGTTGATCGAACCACATACTGCATACATAAGAGAAAAAAATGAATTCCT -1141

ACTTAGATTATAAATATTTTTATTTTAGTTTTGATATATGCAATACGAAAAGAGTAACAA -1081

CTATACTTACTTTCAAAAATAAAATGGACCCCTACAAATATATGGGGGCC**AGAAACAA**AT -1021

**AE-box**

GTTTCATCCTGCTGTACAATAATTGATACAGAAAAACGGCAACGTTCATTGTCAATTTGA -961

ATTTAGTTGTGAGATTCGCGCCGGAAAAATAGAAGTTGAGAGAAGAAGAAGTGTAATTGA -901

ATGCCAAAAGTGATGTAAAAAGTCAGAAAAGAGTTGAAAACTAAAGAGTAATGACAAAAT -841

ATGTTGCGTTTG**CGTACGTCCA**TGAAATTGT**CAACTG**TCAAGTGATCCTTCCAAGGCTTT -781

**ABRE** **MYB,MBS**

GAT**AACGAC**TATCGATCTTTACATTTTGAAAGTA**CATTTG**TTATTAATTACTATGGAGTT -721

**TGA**  **MYC**

TAGAAATCTCACCATTTTA**CATTTGCCGAAA**TATACTTGTTGTATAAATACAAAATTATA -661

**MYC LTR**

CTGTAAATACAAAAAAGAACAGTGTATGCGTATTTTTCCTACTT**TCTTAC**TTTCTTTACA -601

**TCT-motif**

CATTTCTGGTTTTTGTACCCAAAAATAAAAAGCACTTCATGGTTCATACTT**CATTTG**TAC -541

**MYC**

ACTACAGTAACATCCTTATTAAAAAGCGTAAGAAAATGGAATATTGTTTGTGAATTGGGC -481

CGGCCCATCCTTAAGCACAATTTTCGGCCTATTAGAGATTTATGAGACAGAAAAAAAACG -421

AGCGTTAGAATATCGGTATTCGGTAACAAG**AAACCAAACCA**AACCGAATTATATTTGAAC -361

**ARE ARE**

TGGTTCGGTTTGATTTTTTCGAT**TACGGTC**TGATGTAGATTAAATAAAATAATTAAAAAG -301

**ABRE**

TTCCAGAAGCATGCAGAAGAAGATTGAAGAAC**ACCGAG**ATA**CCGAG**ATTTACAGAGGATC -241

**DRE1 DRE1**

AAATCAAAAGCAAAAGAGAAAGAGAGAAGAAGTAGAAGAATCATCATCTTTAAAGC**CAAA** -181

**cir**

**AATATC**AATAAATTAGATTAGAGAATACTTAAGCTTGAAGCAATCTCGTCTTTAGCCTTT -121

**cadial**

ATTGTCGAATTACTAATTAATCTCGAAGCAGAGCACTCTCTGTTCCGATGAATGGATCTC -61

ATCTAAAAAATCACCGTGTCTCTCGTCCCTGGTTTGGTCCTTCGAGAAGTGTGAACAAAA -1

**ATG**

| Motif | **Organism** | **Sequence** | **position** | **Functions** |
| --- | --- | --- | --- | --- |
| ABRE | Arabidopsis thaliana | CACGTG | -1460 | involved in the abscisic acid responsiveness |
|  | Hordeum vulgare | CGTACGTGCA | -819 |  |
|  | Arabidopsis thaliana | TACGGTC | -331 |  |
| AE-box | Arabidopsis thaliana | AGAAACAA | -1023 | part of a module for light response |
| ARE | Zea mays | AAACCA | -385 | essential for the anaerobic induction |
|  |  | AAACCA | -380 |  |
| circadian | Lycopersicon esculentum | CAAAGATATC | -175 | cis-acting regulatory element involved in circadian control |
| DRE1 | Zea mays | ACCGAGA | -263 | dehydration-responsive element |
|  |  | ACCGAGA | -255 |  |
| G-box | Arabidopsis thaliana | CACGTG | -1460 | involved in light responsiveness |
| LTR | Hordeum vulgare | CCGAAA | -690 | involved in low-temperature responsiveness |
| MBS | Arabidopsis thaliana | CAACTG | -804 | involved in drought-inducibility |
| MYB | Arabidopsis thaliana | CAACAG | -1578 | plant development and responses to stresses |
|  |  | TAACTG | -1425 |  |
|  |  | TAACCA | -1346 |  |
|  |  | CAACCA | -1307 |  |
|  |  | CAACTG | -804 |  |
| MYC | Arabidopsis thaliana | CATTTG | -741 | different functions in stress resistance, growth, and development |
|  |  | CATTTG | -696 |  |
|  |  | CATTTG | -544 |  |
| TCT-motif | Arabidopsis thaliana | TCTTAC | -611 | part of a light responsive element |
| TGA-element | Brassica oleracea | AACGAC | -772 | auxin-responsive element |
