# Supplementary material for: SYNAPTOTAGMIN 4 is expressed mainly in the phloem and participates in abiotic stress tolerance in Arabidopsis
Source: Front Plant Sci. 2024 Jul 1;15:1363555. doi: 10.3389/fpls.2024.1363555 (PMC11246894; doi:10.3389/fpls.2024.1363555)
Supplement: Supplementary file 3 [file Table_3.docx]

**Supplementary Table 3:** Positions of T-DNA insertions and *SYT4*-T-DNA junction sequences in *syt4* alleles

Coding AtSYT4 sequences are shown in capital letters, intron sequences in lowercase letters, DNA filler sequences in italic bold lowercase letters, T-DNA sequences in capital bold letters and a common sequence for genomic and insert DNA in underlined letters. Arrows with numbers indicate insert incorporation sites in the *AtSYT4* genomic sequence.

**1. SALK_201787 (*syt4-1*)**


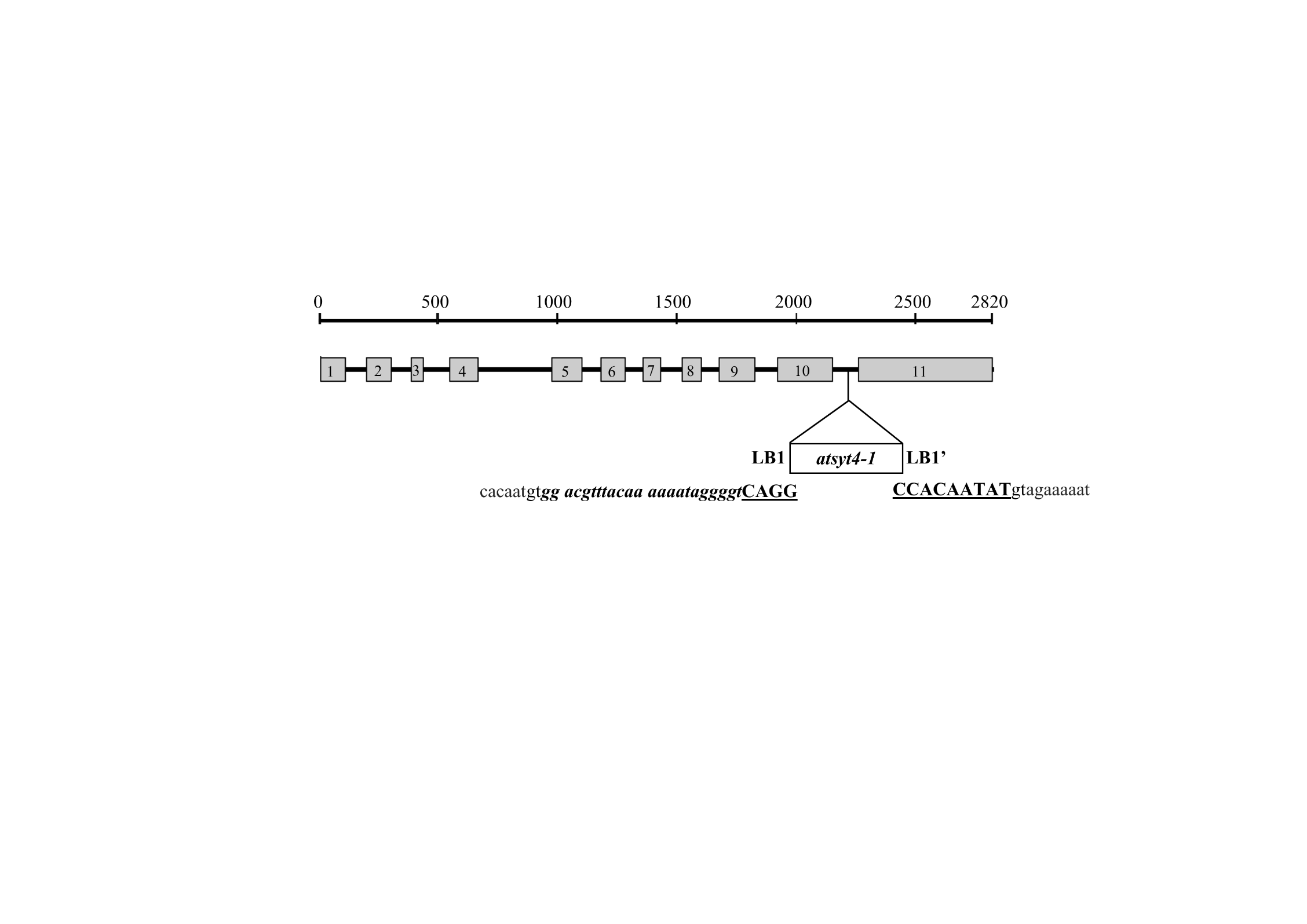


2101 AGTCAAGGAT TTAGAGATCC AAAGGGATAC AAAAAATAGG GGTCAGgtta gtatttcctc intron 10

2161 ctatcataac tattgtttcc atattcataa cacaatgt***gg acgtttacaa aaaatagggg***

***t*CAGGATATA** ……………**T-DNA**
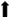


**LB1** 2198

**T-DNA…………… GACGCTTAGA GTTTACACCA CAATAT**gtag aaaaatataa catatccatt

**LB1’**
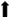


2196

2221 ttagttagga ctataaaaga tatatttgtg gtgatatagG TGCAGTTGGA GCTCTTATAT exon 11

**2. SAIL_652_E05 line (*syt4-2*)**

**
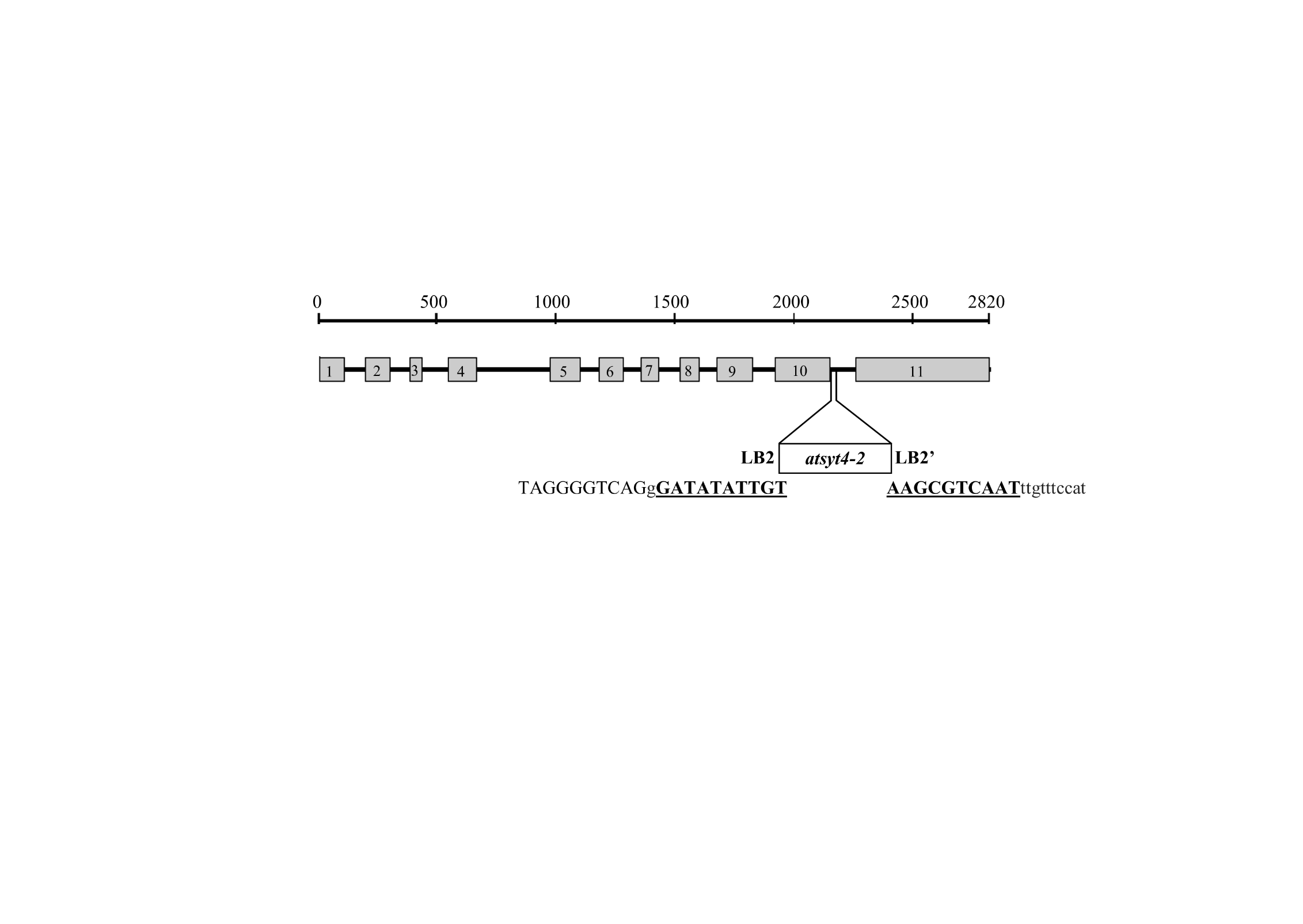
**

2041 TGCTCAAGTA CCGTTAAATG AACTCGTACC CGGGAAAGTT AAAGATATTT GGTTGAAGTT exon 10

2101 AGTCAAGGAT TTAGAGATCC AAAGGGATAC AAAAAATAGG GGTCAGg**GAT ATATTGTGGT**

**GTAAACAAAT ……………T-DNA
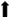
**

**LB2** 2147

**T-DNA……………** **ATTAAGTTGT**

**CTAAGCGTCA** **AT**ttgtttcc atattcataa cacaatgtag aaaaatataa catatccatt intron 10

**LB2’
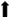
**

2172

2221 ttagttagga ctataaaaga tatatttgtg gtgatatagG TGCAGTTGGA GCTCTTATAT exon 11

**Deleted sequence from *SYT4***: ttagtatttcctcctatcataacta

**3. SAIL_359_H05 line(*syt4-3*)**


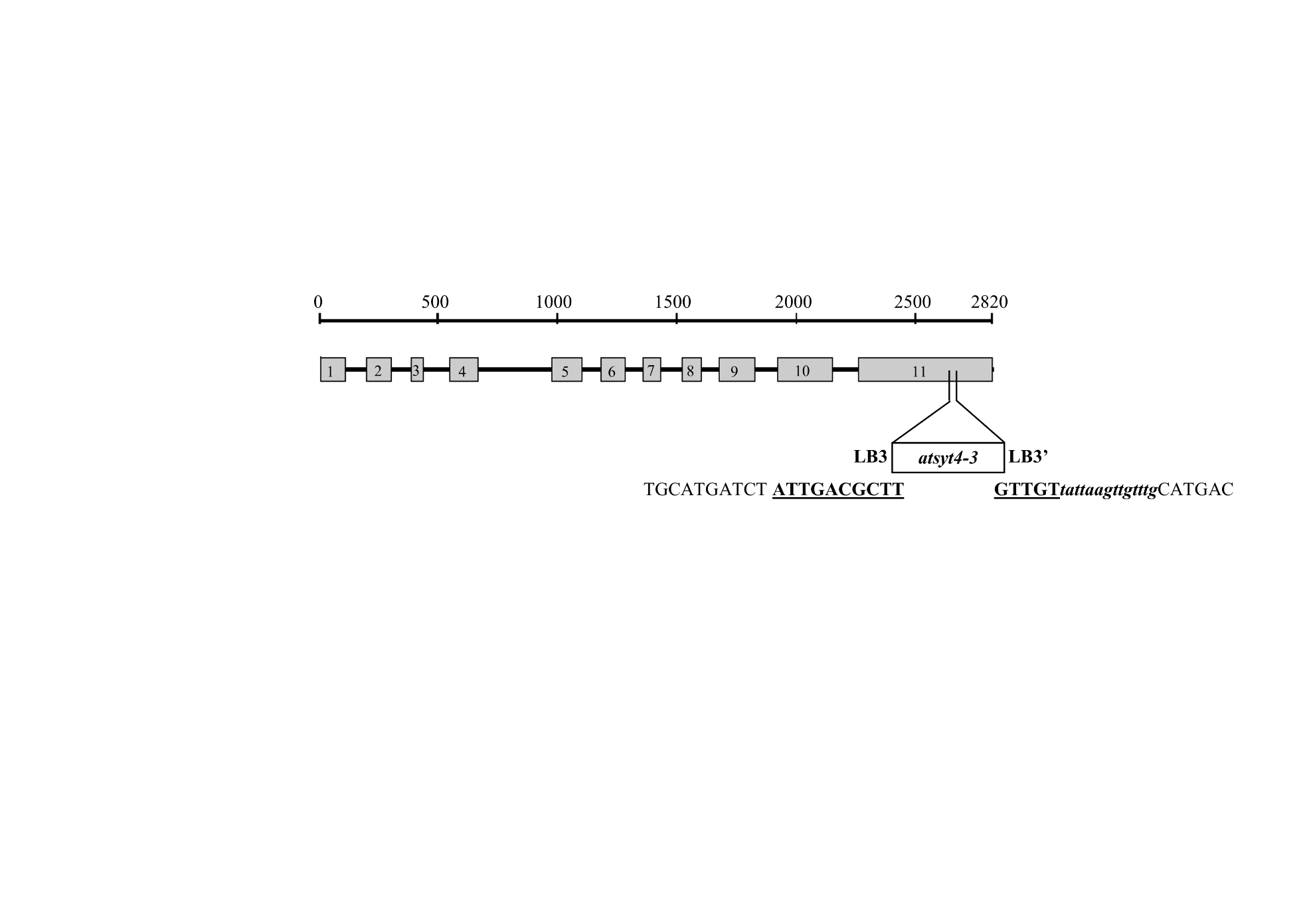


2521 ACATTGAAGA AATCAGAAAC GAAATCCAAG ACAAGGGTAG TACCGGATAG CTTGAATCCG exon 11

2581 GTTTGGAACC AGACGTTTGA TTTTGTAGTG GAAGATGCTT TGCATGATCT ATTGACGCTT

**AGACAACTT AATAACACAT** ……………**T-DNA**
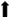


**LB3** 2630

**T-DNA**…………… **AAACGTCCGC** **AATGTGTTAT** **TAAGTTGT*ta***

**LB3’**

***ttaagttgtt*** ***tg***CATGACAA ATTTGGCAAG GACAAGATAG GGAGAGTGAT AATGACATTG


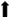


2651

2701 ACGCGAGTGA TGTTAGAAGG AGAGTTTCAA GAGTGGTTTG AGTTAGATGG AGCTAAATCA exon 11

2761 GGGAAGCTTT GTGTCCATCT TAAATGGACT CCTAGGCTTA AGCTCAGAGA CGCCTCTTGA

**Deleted sequence *from SYT4***: GAAGTATGGGAC

**4. GABI-215E11 line(*syt4-4*)**


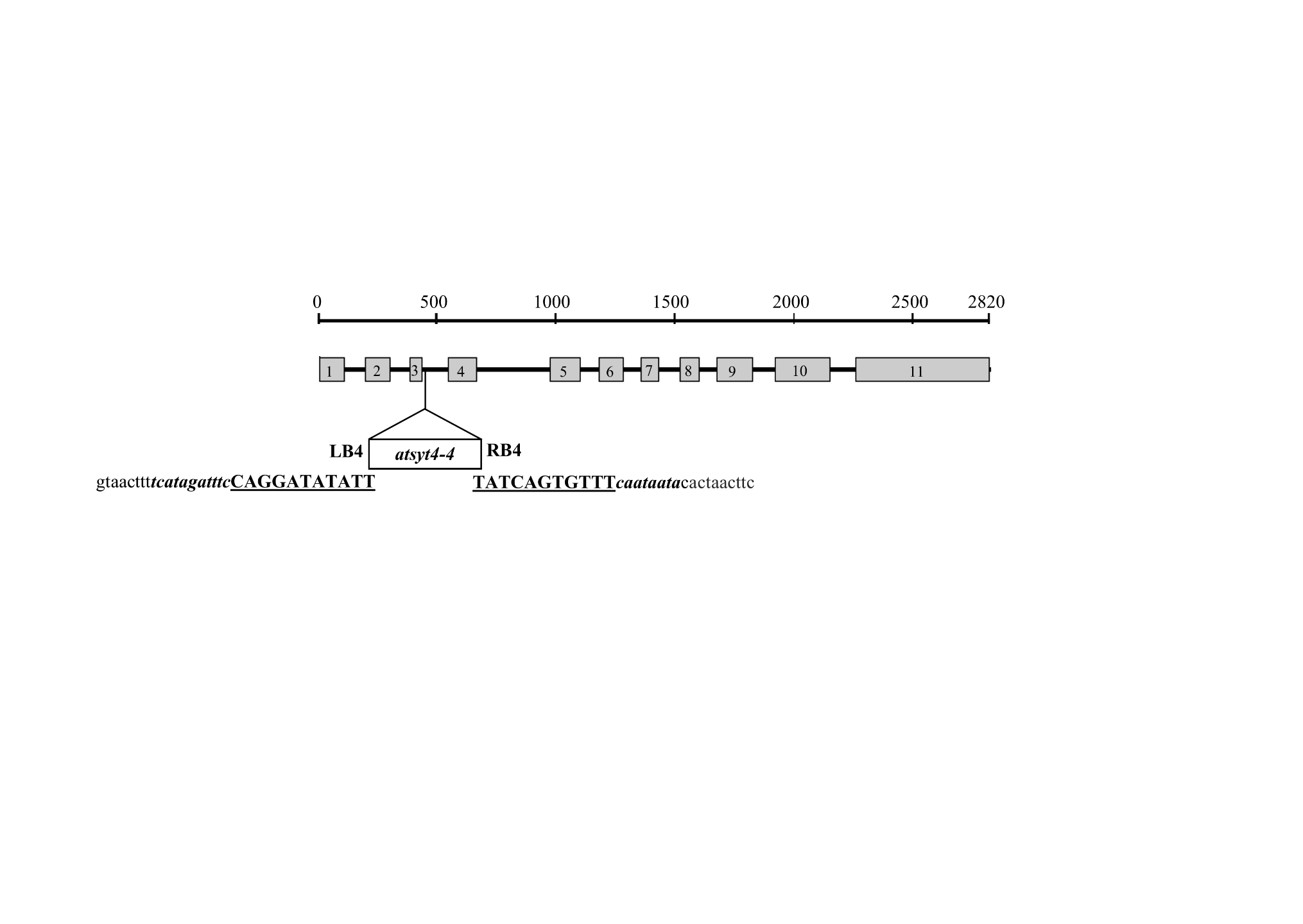


361 attgatactt gtccttgatt aaactacagT TAAATTGGCT TAATCTTGAA CTTGAAAAGA exon 3

421 TCTGGCCTTA TGTAAATGAG gtaacttt***tc*** ***atagatttc*C** **AGGATATATT** **CAATTGTAAA TGGCTCATGT ……………T-DNA**
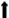


448

**T-DNA…………… CGCCTTCGGT**

**TTAAACTATC AGTGTTT*caa* *taata***cacta acttcgtttt tctgttggtt aatcttgtag

**
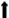
**

506

541 GCAGCTTCTG AGCTGATTAA AAGCAGTGTG GAACCAGTAC TTGAACAGTA TACACCAGCT exon 4

**Deleted sequence from *SYT4***: aattaacacgcaatggtactttttttttttacctttttgg atgtgttgatatatctt

**5. GK668A12 line(*syt4-5*)**

**
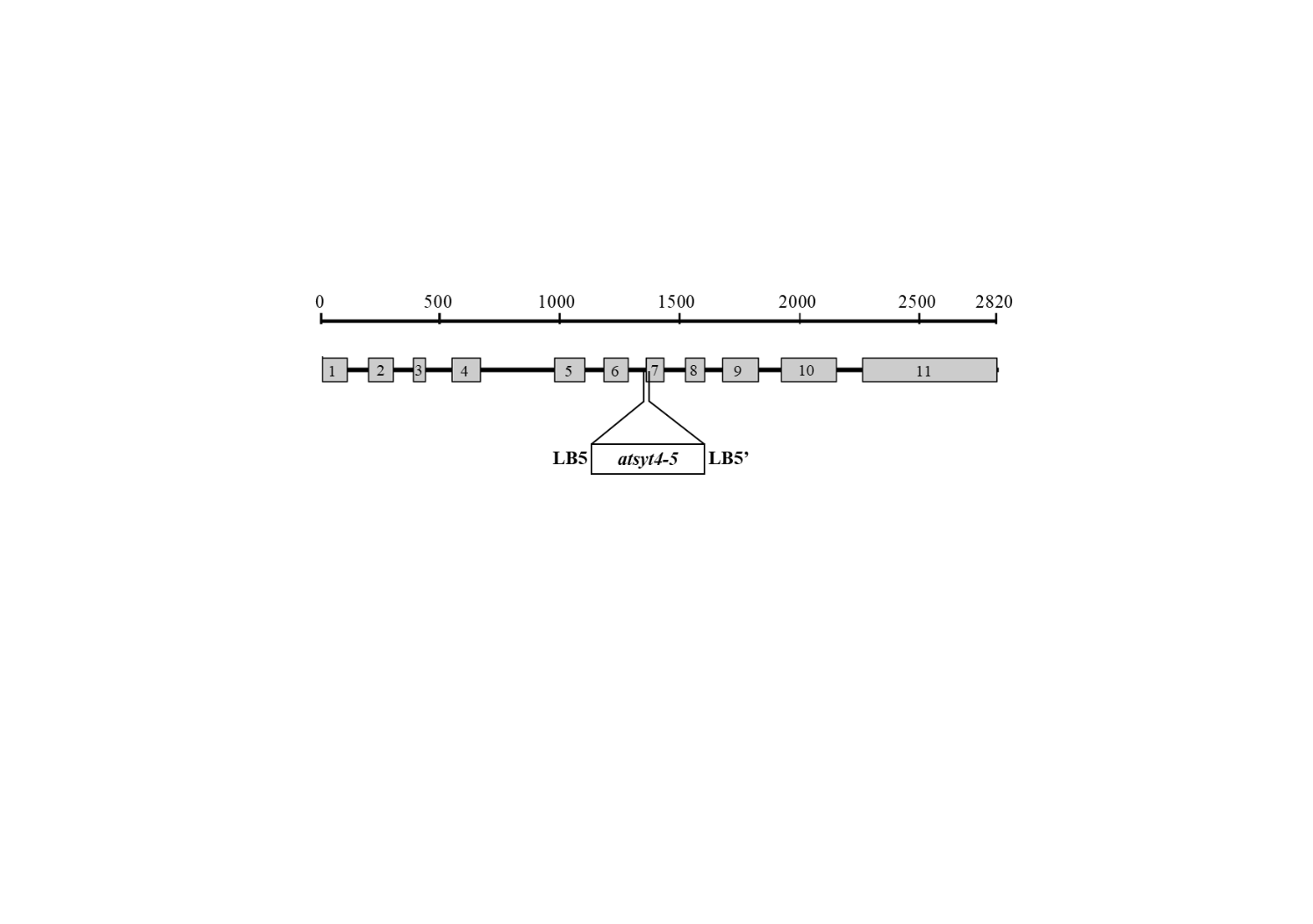
**

1261 ATTCACTCCG CGAGAAGgta acttagtgat gatatgatat atattgcttg ttataataac intron 6

1321 ttagtaatct tctcttgttt tctgtg***ga*CA GGATATATTC CAATTGTAAA ……………T-DNA**

**
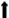
**

1346

**T-DNA………… CCGGACATGA AGCCATTTA*ac* *aattttccat*** ATTTTACCCT CAAAGTTAT

**
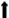
**

1362

1381 AGGTGGTGAA TTAACATCAA TTCCAGGGAT TTCAGATGCA ATCGAAgtat gccaaaaacc intron7

**Deleted sequence from *SYT4***: agcagAAAGGGCTTG
