# Supplementary figures and images for: SYNAPTOTAGMIN 4 is expressed mainly in the phloem and participates in abiotic stress tolerance in Arabidopsis
Source: Front Plant Sci. 2024 Jul 1;15:1363555. doi: 10.3389/fpls.2024.1363555 (PMC11246894; doi:10.3389/fpls.2024.1363555)

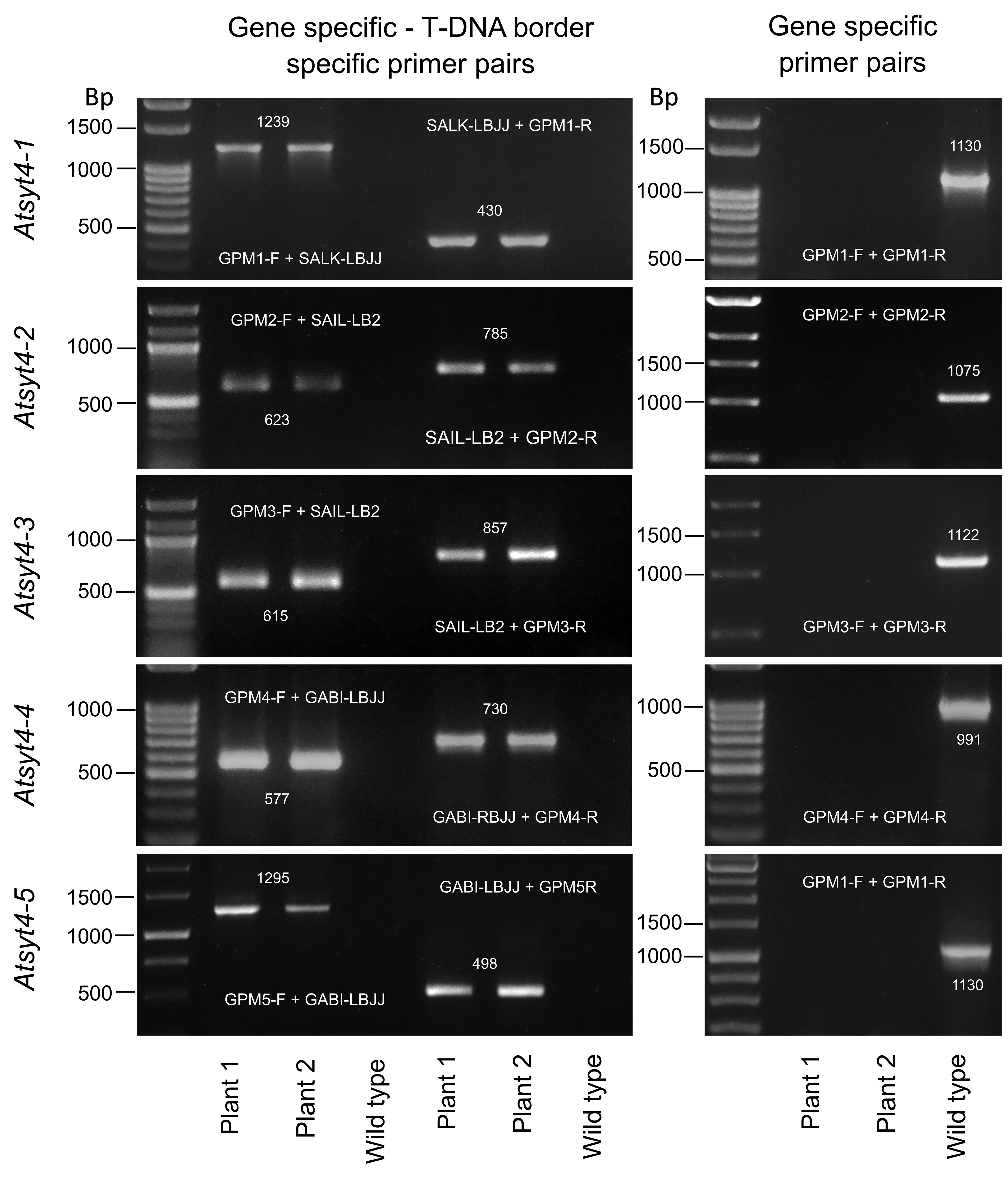

Supplement: Supplementary Figure 1 — Confirmation of the presence of T-DNA inserts and homozygosity of syt4 alleles. [file Image_1.tif]

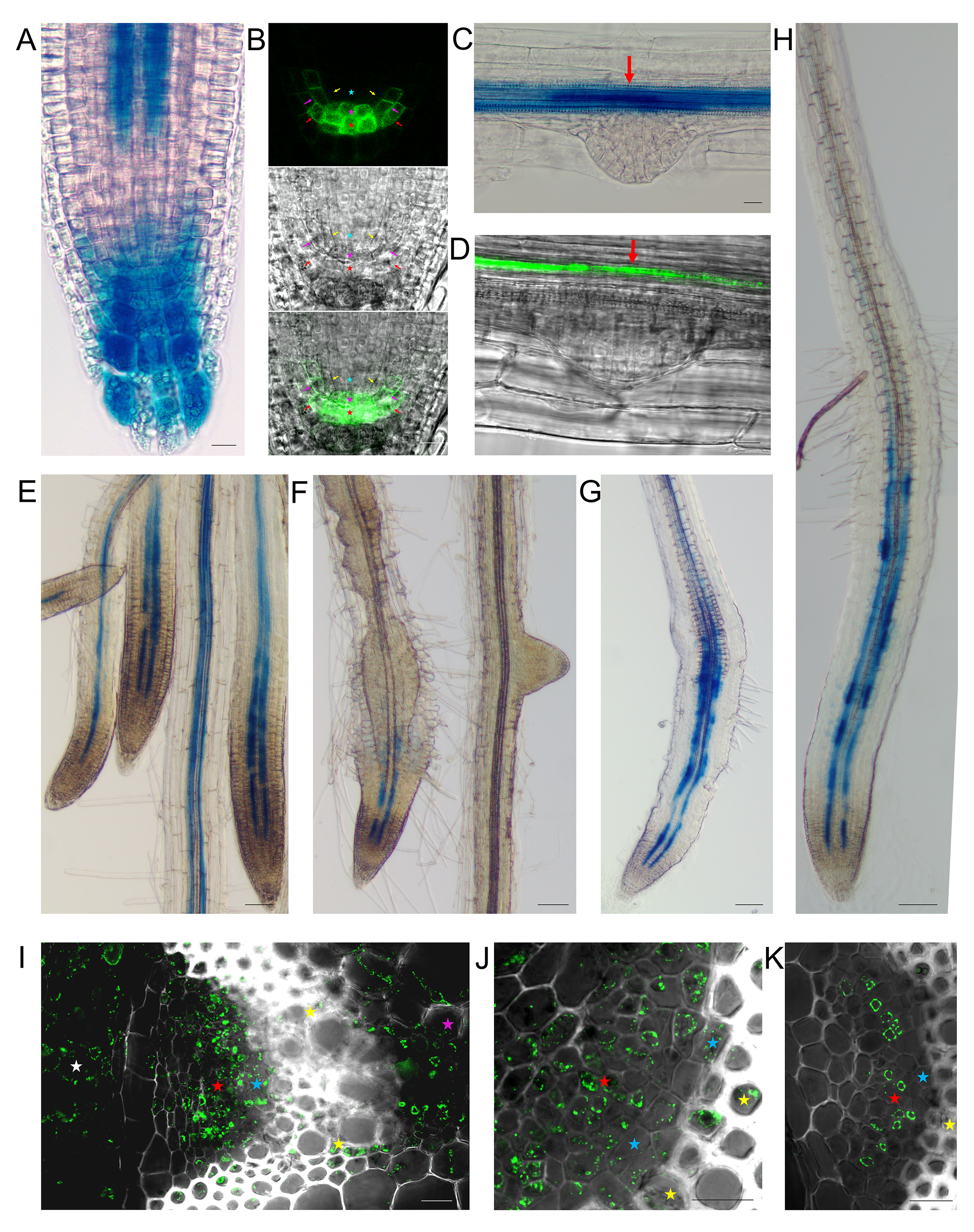

Supplement: Supplementary Figure 2 — Analysis of the SYT4 promoter activity and SYT4 and SYT1 protein distribution. The root with the strong SYT4 promoter activity in the RC is in (A). (B) shows the distribution of SYT4 protein in the root stem cell niche (red asterisk marks columella initials, purple QC, cyan stele initials, red arrows point to epidermis/lateral root cap initials, purple to endodermis/cortex initials and yellow to pericycle initials). (C) shows the activity of the SYT4 promoter and (D) SYT4 protein pattern in the root region with a lateral root primordium (arrows). (E) shows SYT4 promotor activity in root tips of 10 days seedling growing on SCM. In (F), a seedling was grown for the last 2 days on SCM supplemented with 1 µM 2,4-D, (G) with 300 mM mannitol and (H) with 150 mM NaCl. The section through the shoot of the SYT1-Dendra2 plant documenting the occurrence of SYT1 protein in all tissues is shown in (I) (white asterisk marks cortex, red phloem, blue cambium, yellow xylem, violet pith). The close view of the phloem region is in (J). (K) shows the phloem region of the SYT4-GFP shoot (asterisks in J and K are as in I). Size bars – (A–D, I–K) = 20 µm; (E–H) = 100 µm. [file Image_2.tif]
